# Persistence of Association of Blood Biomarkers With Risk of Recurrent Stroke on Long-Term Follow-Up: Population-Based Study

**DOI:** 10.1161/STROKEAHA.126.056325

**Published:** 2026-08-03

**Authors:** Linxin Li, Annette I. Burgess, Julia Duerden, Katherine Onions, Helen C. Segal, Peter M. Rothwell

**Affiliations:** Nuffield Department of Clinical Neurosciences, Wolfson Centre For Prevention of Stroke and Dementia, John Radcliffe Hospital, University of Oxford, United Kingdom.

**Keywords:** biomarker, ischemic attack, transient, risk assessment, secondary prevention, stroke

## Abstract

**BACKGROUND:**

Raised inflammatory biomarkers predict an increased short-term risk of recurrent vascular events after stroke or transient ischemic attack, but it is uncertain whether the hazard is maintained thereafter. In the absence of previous population-based studies, we aimed to determine the predictive value of biomarkers for the long-term risk of recurrent stroke in intensively treated patients following transient ischemic attack or stroke.

**METHODS:**

We studied 12 blood biomarkers related to inflammation, neuronal cell damage, and thrombosis soon after transient ischemic attack or stroke in a population-based study (OXVASC [Oxford Vascular Study]; 2002–2011) with follow-up to 2025. We used Cox and negative binomial regressions to assess associations between each log-transformed biomarker (per SD increase) and recurrent stroke (first event and total number), adjusted for age, sex, and baseline vascular risk factors.

**RESULTS:**

Of 1292 consecutive patients (mean/SD age, 73.1/13.2 years; 47.8% male), 365 recurrent strokes occurred in 280 patients during 11 336 patient-years. All inflammatory biomarkers were associated with 10-year risk of recurrence, with little impact of adjustment for other risk factors (adjusted incidence rate ratio: IL-6 [interleukin-6], 1.21 [95% CI, 1.04–1.42]; CRP [C-reactive protein], 1.29 [95% CI, 1.11–1.49]; TNFR-1 [tumor necrosis factor receptor-1], 1.24 [95% CI, 1.05–1.46]; and NGAL [neutrophil gelatinase–associated lipocalin], 1.15 [95% CI, 0.99–1.33]). Moreover, number of elevated (top quartile) inflammatory markers was associated with higher burden of recurrence (incidence rate ratio, 1.31 [95% CI, 1.15–1.50]; *P*<0.001), with no diminution over time (90 days: hazard ratio, 1.24 [95% CI, 0.98–1.57]; 90 days–1 year: 1.24 [95% CI, 0.95–1.62]; 1–5 years: 1.22 [95% CI, 1.01–1.47]; and 5–10 years: 1.34 [95% CI, 1.06–1.68]), and similar associations for transient ischemic attack and minor stroke. Biomarkers related to neuronal cell damage or thrombosis were generally less predictive of recurrent stroke.

**CONCLUSIONS:**

Inflammatory biomarkers were more predictive of long-term recurrent stroke than neuronal or thrombotic biomarkers, particularly when several inflammatory markers were raised. Predictive value was independent of other vascular risk factors and did not diminish with duration of follow-up, supporting trials of anti-inflammatory strategies for long-term secondary prevention.

Stroke and recurrent vascular events are a major cause of disability and death within an aging population.^1^ Although secondary prevention in transient ischemic attack (TIA) and stroke reduces early recurrent stroke by up to 80%,^2^ and anticoagulation reduces atrial fibrillation-related stroke risk by up to 60%,^3^ there is still substantial residual risk of later recurrence in patients on optimal secondary prevention treatment.^4,5^ Although more intensive secondary prevention strategies are available, such as the additional use of PCSK-9 (proprotein convertase subtilisin/kexin type 9) inhibitors or anti-inflammatory therapy with canakinumab,^6,7^ they might only be cost-effective in subgroups with high risk of recurrence.

Traditionally, the prediction of the long-term individual risk for a recurrent stroke is based on many clinical variables, but such models remain imperfect.^8,9^ Large cohort studies have related biomarkers of inflammation, thrombosis, or neuronal function to stroke risk stratification in primary prevention,^10–13^ but it is unclear if the evidence from these studies is generalizable in secondary prevention when patients are on optimal medical therapy. Indeed, biomarker studies in secondary prevention have generated conflicting results,^14–19^ and most of these studies were hospital-based with short follow-up, typically up to 1 year.^15,17–19^ A recent individual participant data collaboration showed convincing evidence that inflammatory biomarkers, such as IL-6 (interleukin-6) or hsCRP (high-sensitivity C-reactive protein), were associated with poststroke vascular events,^20,21^ but only one old study (1998–2003) included had follow-up to 10 years,^16^ and there was no analysis of how the predictive value of biomarkers evolved with time. Moreover, previous studies have focused on the predictive value of biomarkers for the first recurrent event. However, most patients with TIA or stroke tend to survive for many years after presentation, and many experience multiple recurrences. Identifying biomarkers that are potentially associated with long-term cumulative burden of recurrent stroke may, therefore, better inform risk stratification and guide more intensive secondary prevention strategies.

In the absence of similar studies, we aimed to assess in a large prospective population-based study, whether a panel of biomarkers related to inflammation, thrombosis, and neuronal function, chosen based on evidence of possible prognostic value from previous studies of cardiovascular disease and stroke,^18,22–27^ had predictive value for the 10-year risk of recurrent stroke (first event and total number of events) in unselected patients with TIA or stroke on current best medical therapy.

## Methods

The study was reported according to the STROBE reporting guidelines (Strengthening the Reporting of Observational studies in Epidemiology). The data that support the findings of this study will be considered by the corresponding author upon reasonable request.

The OXVASC (Oxford Vascular Study) is an ongoing population-based study of the incidence of all acute vascular events, including TIA, strokes, acute coronary syndromes, and peripheral vascular events.^28^ The study population comprises all 92 728 individuals, irrespective of age, registered with 8 general practices in Oxfordshire, United Kingdom. The multiple overlapping methods used to achieve near complete ascertainment of all individuals with TIA or ischemic stroke have been reported previously.^28^ This analysis includes consecutive adult patients (≥18 years) with a first TIA or stroke ascertained between April 1, 2002, and March 31, 2011, with follow-up until March 31, 2025.

Patients were assessed by study physicians as soon as possible after the initial presentation. Demographic data, premorbid use of preventive medication, and premorbid risk factors were collected from face-to-face interviews and cross-referenced with general practice records. Patients routinely had brain imaging (computed tomography or magnetic resonance imaging), vascular imaging (carotid Doppler or computed tomography angiography/magnetic resonance imaging angiography or digital subtraction angiography), 12-lead electrocardiography, and routine bloods (full blood count, clotting, CRP [C-reactive protein], erythrocyte sedimentation rate, liver function, renal function, thyroid function, electrolytes, and lipid profile). Holter, transthoracic echocardiogram, thrombophilia, and vasculitis screening, as well as genetic tests where appropriate, were performed for cryptogenic TIA/stroke. All cases were reviewed by a senior neurologist, and stroke cause was classified according to the modified TOAST (Trial of ORG 10172 in Acute Stroke Treatment) criteria.^29^ Severity of events was measured using the National Institutes of Health Stroke Scale score. Events were classified as minor stroke if there was a focal neurological deficit lasting >24 hours and a National Institutes of Health Stroke Scale score ≤3 at the time of assessment by a study physician.^2^

All patients were followed up face to face at 1, 3, and 6 months, and 1, 5, 10, and 20 years by a study nurse or physician to determine recurrent strokes. For patients who had moved out of the study area, telephone follow-up was performed. All deaths during follow-up were recorded with causes. All recurrent vascular events, including stroke and myocardial infarction that presented to medical attention, would also be identified prospectively by ongoing daily case-ascertainment. If a recurrent vascular event was suspected at a follow-up visit or referred by the general practices to clinic or admitted, the patient was reassessed and investigated by a study physician.

All patients were started on appropriate secondary preventive treatment on the day of the initial clinic assessment or after hospital admission, which generally included antithrombotic treatment, a statin, and antihypertensive medication according to the EXPRESS study (Early Use of Existing Preventative Strategies for Stroke) protocol.^2^ Antithrombotic treatment was aspirin (300-mg loading dose followed by 75 mg daily) in all cases not requiring anticoagulation, with the addition of clopidogrel (300-mg loading dose followed by 75 mg daily) in patients with an ABCD score ≥4 or with recently symptomatic large artery stenosis.^2^

Nonfasting blood samples were taken at the time of first assessment and included serum and 3.2% buffered trisodium citrate plasma and lithium heparin plasma (Vacutainer tubes [Becton Dickinson, United Kingdom]). Samples were centrifuged at 3000*g* for 10 minutes, and aliquots of serum and plasma were stored at –80 °C before analysis when they were thawed for use at 37 °C  for 10 minutes. All times from sampling to freezing were documented, typically within 4 hours of taking.

### Assay Methods

We included inflammatory biomarkers: IL-6, CRP, TNFR-1 (tumor necrosis factor receptor-1), and NGAL (neutrophil gelatinase–associated lipocalin); markers of neuronal function and injury: NSE (neuron-specific enolase) and BDNF (brain-derived neurotrophic factor); and thrombotic biomarkers: TM (thrombomodulin), fibrinogen, P-selectin (platelet selectin), D-dimer, VWF (von Willebrand Factor) antigen, and PZ (protein Z).

The assay methods and their intracoefficients and intercoefficients of variation were reported previously (Table S1).^30,31^ Briefly, a Randox Evidence Investigator Biochip multiple immunoassay system (Randox Laboratories Ltd, County Antrim, United Kingdom) using lithium heparin plasma simultaneously measured biomarkers via 2 panels: cerebral array I for IL-6 and BDNF, and cerebral array II for CRP, NGAL, TNFR-1, NSE, and TM. Thrombotic markers were assayed using citrated plasma for fibrinogen, D-dimer, and VWF antigen by immunoturbidometric assays (Sta-Liatest VWF and Sta-Liatest D-Dimer, Diagnostica Stago, Asniers, France) using an automated coagulation analyzer STA (Diagnostica Stago). Commercial ELISA kits were used to measure human P-selectin (P-sel, R&D Systems, United Kingdom) and PZ (Diagnostica Stago).

For thrombotic markers, we excluded cases with limited volume and those who were on concurrent anticoagulant therapy.

### Statistical Analysis

We performed complete case analyses without any imputation for missing data.

We compared the frequency of risk factors and levels of different biomarkers in those with recurrent stroke versus those without using the χ^2^ test for categorical variables, the *t* test for continuous variables that were normally distributed, and the Mann-Whitney *U* test for continuous variables that were skewed. Normality of continuous variables was assessed using graphical methods (histograms and Q-Q plots) and the Shapiro-Wilk test. With the exception of VWF, fibrinogen, and PZ, which were normally distributed, all biomarkers were log-transformed. Cox proportional hazard models were used to assess risks of first recurrent stroke per SD increase of each biomarker. Analyses using per quartile increase or top versus bottom quartile were also performed. Associations of biomarkers with recurrent stroke were calculated unadjusted and adjusted for age and sex (model 1) and adjusted for age, sex, and baseline vascular risk factors (model 2). Additional adjustment for the delay in sampling was also performed. We also assessed the associations of individual biomarkers at risks of recurrent stroke at different time points, censoring for recurrent events during that time point (ie, <90 days, 90 days–1 year, 1–5 years, and 5–10 years). Negative binomial regression was also used to assess the predictive values of the above biomarkers in predicting the overall burden of recurrent stroke, which is measured by the total number of recurrent strokes during follow-up. Bonferroni adjustment for multiple comparisons (based on 12 biomarkers tested) was applied.

In addition to the primary outcome including recurrent stroke, we also assessed risks of a composite outcome of recurrent stroke, myocardial infarction, and sudden cardiac death.

In addition to individual biomarkers, patients were also grouped according to the number of biomarkers that were in the top quartiles, and we compared risks of recurrent stroke by the number of biomarkers in the top quartile for inflammatory and thrombotic markers, respectively, using Cox regression and negative binomial regression adjusted for age, sex, and vascular risk factors. As there were only 2 markers for neuronal function, we did not apply this approach to this category.

Analyses were also stratified by history of coronary artery disease and TOAST subtypes. Sensitivity analyses excluding major stroke, early recurrence in the first 90 days, or excluding measurements taken within the first 24 hours were also performed.

All analyses were performed using IBM SPSS, version 29, and Stata 15.

## Results

Of 1292 consecutive patients included for the analysis, 972 (75.2%) were TIA or minor stroke. The mean/SD age was 73.1/13.2 years, and 617 (47.8%) were male. Baseline characteristics of all included patients were presented in Table 1. The median delay from onset of event to sampling was 5 (interquartile range, 3–11) days. A total of 1207 samples (401 TIA and 806 stroke) were assayed for inflammatory markers and markers of neuron cell function; 79 missing values were due to sample volume constraints, and there were additional 6 assay failures. Thrombotic markers were measured in a maximum of 1072 patients after excluding cases with limited volume (n=152) and concurrent anticoagulant therapy (n=68). Overall, there was good cross-correlation within the subset of inflammatory markers (Table S2). In addition, fibrinogen, VWF, and D-Dimer correlated modestly but statistically significant with each other and the inflammatory markers.

**Table 1.**
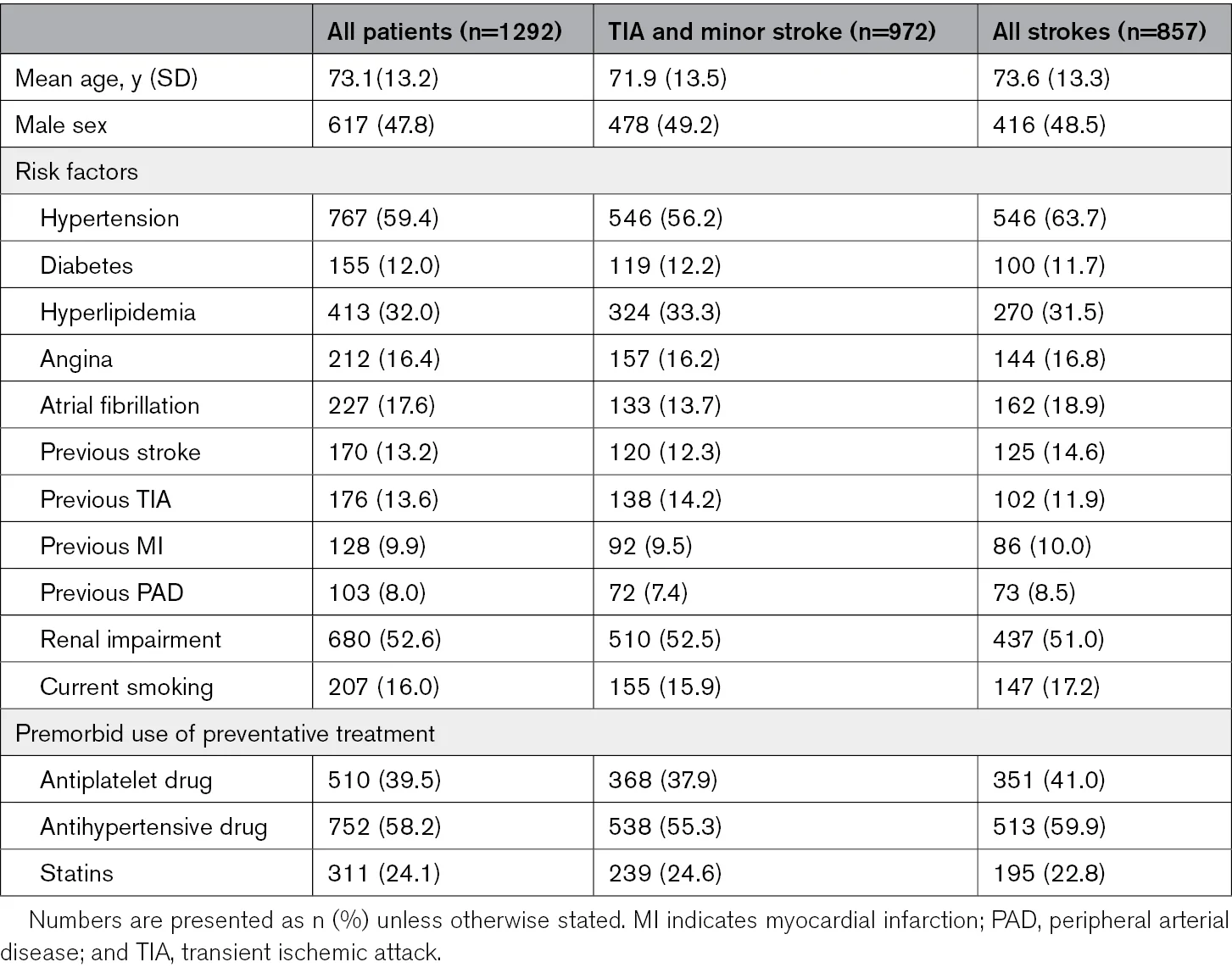
Baseline Characteristics of All Patients With Suspected TIA or Stroke in the Oxford Vascular Study

During 11 336 patient-years of follow-up (median follow-up, 8.9 years), there were 365 recurrent strokes among 280 patients (first recurrence type was 255 ischemic and 25 hemorrhagic). Patients who had recurrent strokes were older, had a higher prevalence of known risk factors, such as hypertension, atrial fibrillation, and previous stroke, and were more likely to be on premorbid preventative treatment (Table 2). Baseline levels of the measured biomarkers were not statistically different between those with recurrent stroke versus those without, apart from D-dimer (Table 2), although the significance was lost if corrected for multiple comparisons.

**Table 2.**
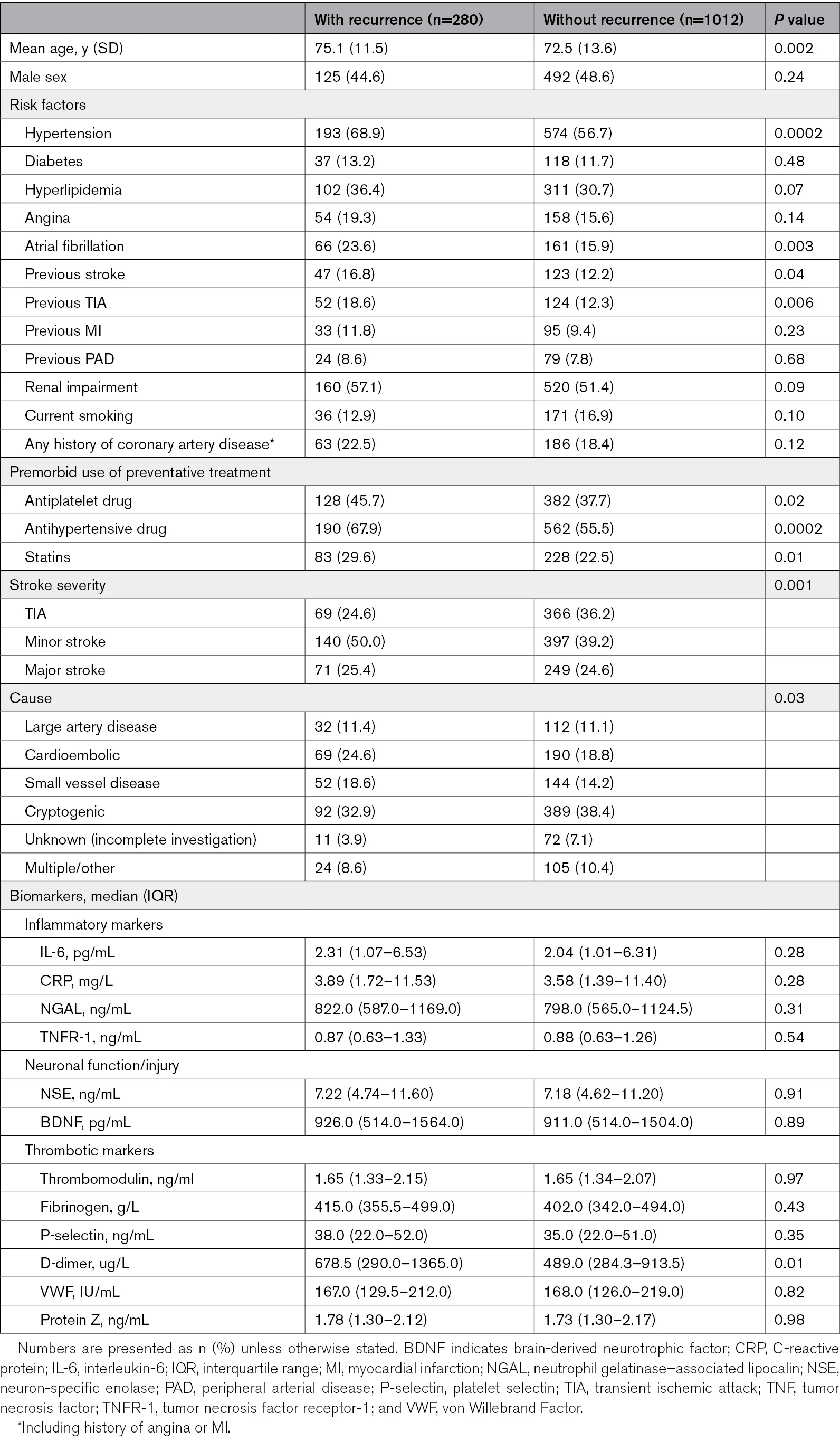
Baseline Characteristics and Levels of Studied Biomarkers of Patients With TIA and Stroke Stratified Into Those With Recurrent Stroke Versus Those Without

In unadjusted analyses, several biomarkers were associated with long-term risks of recurrent stroke (Table 3), but the associations were attenuated after adjustment for age and sex, and were further attenuated after adjustment for vascular risk factors (Table 3). The biomarkers that were independently associated with long-term risk of recurrent stroke were CRP (adjusted hazard ratio, 1.20 [95% CI, 1.05–1.37]; *P*=0.006) and D-dimer (1.21 [95% CI, 1.02–1.44]; *P*=0.03), which were also strongest in those with history of coronary artery disease (Table S3), although the significance became borderline if corrected for multiple comparisons (*P*=0.07 and *P*=0.36). Including all recurrent strokes during follow-up increased the statistical power, and some additional inflammatory biomarkers with borderline significance using the time-to-first-event approach became significant (adjusted incidence rate ratio: IL-6, 1.21 [95% CI, 1.04–1.42]; TNFR-1, 1.24 [95% CI, 1.05–1.46]; Table 3). Results were broadly consistent after excluding recurrent events in the first 90 days (Table 3) although the association of CRP and recurrent stroke was no longer significant. In the time-course analysis, CRP was more strongly associated with short-term risk of recurrent stroke, and D-dimer was more strongly associated with recurrence beyond 90 days (Table S4). Results were also consistent in sensitivity analyses including only TIA or minor stroke (Tables S5 and S6) or including all strokes (Tables S7 and S8) and when per quartile change or top versus bottom quartile was used (Table S9). Moreover, adjusting for delay to sampling or excluding measurements taken within the first 24 hours had no meaningful impact on the results (Table S10). For the composite outcome of recurrent stroke, myocardial infarction, or sudden cardiac death, the results were consistent (Table S11) although, contrary to risk of recurrent stroke, no biomarker was significantly associated with long-term risks of myocardial infarction or sudden cardiac death (Table S12), even in those with a known history of coronary artery disease (Table S12).

**Table 3.**
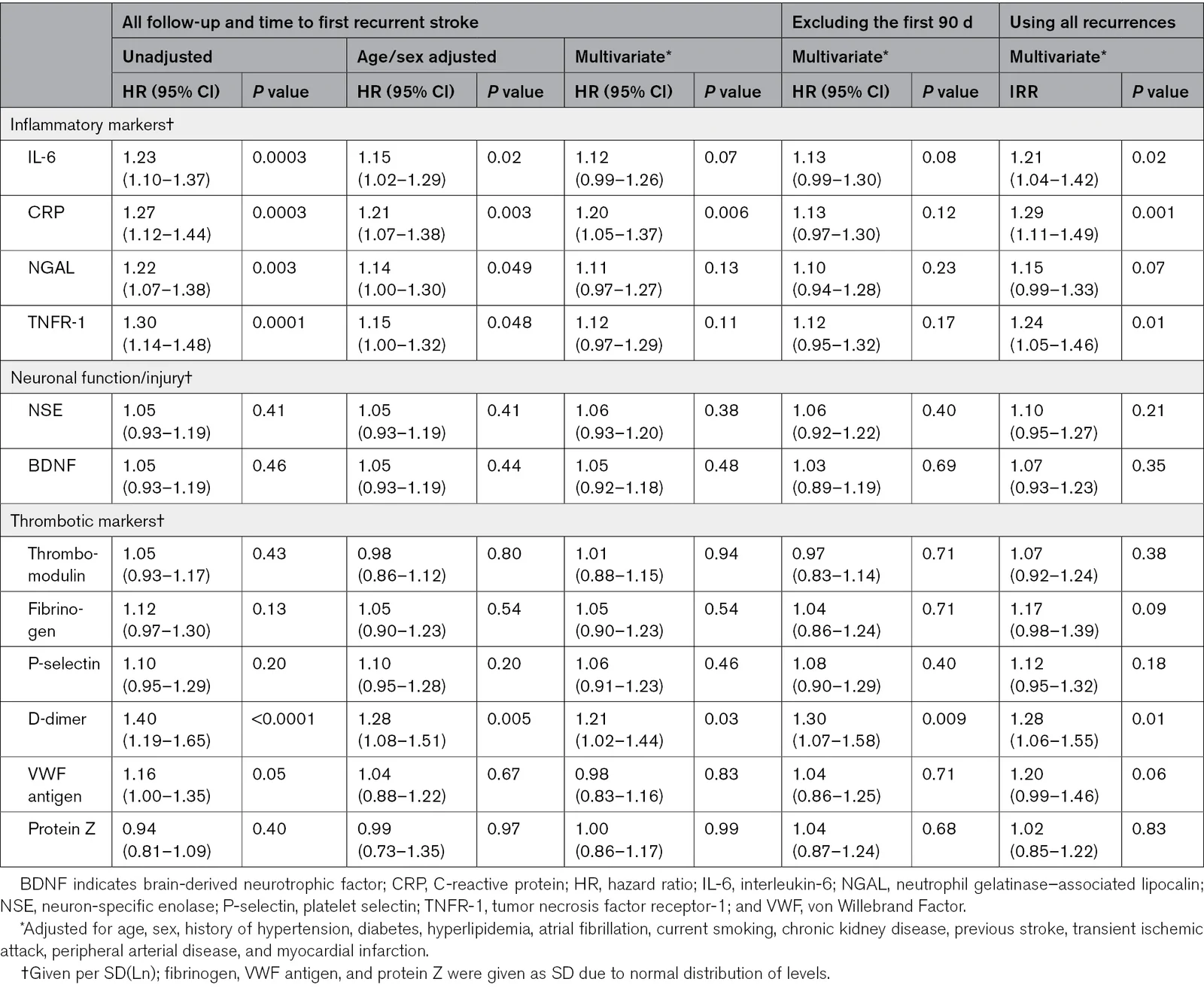
Univariate and Multivariate Analyses of the Associations (Hazard Ratio per SD) of Individual Biomarkers With Long-Term Risk of Recurrent Stroke

We also looked at the associations of biomarkers and recurrent stroke stratified by stroke cause (Figure 1; Tables S13 and S14; Figure S1). Although there was some difference by stroke subtypes with D-dimer being associated with recurrent stroke in cryptogenic events (1.38 [95% CI, 1.00–1.93]; *P*=0.05), the significance was lost after adjusting for multiple comparisons (*P*=0.60). There was also a trend of inflammatory markers being mostly strongly associated with recurrent stroke in large artery disease (Figure 1), which was significant if further stratified by known history of coronary artery disease (IL-6: adjusted hazard ratio, 1.28 [95% CI, 1.06–1.56]; *P*=0.01; CRP, 1.37 [95% CI, 1.06–1.77]; *P*=0.02; NGAL, 1.35 [95% CI, 1.02–1.78]; *P*=0.04; and TNFR-1, 1.35 [95% CI, 1.06–1.73]; *P*=0.02).

**Figure 1.**
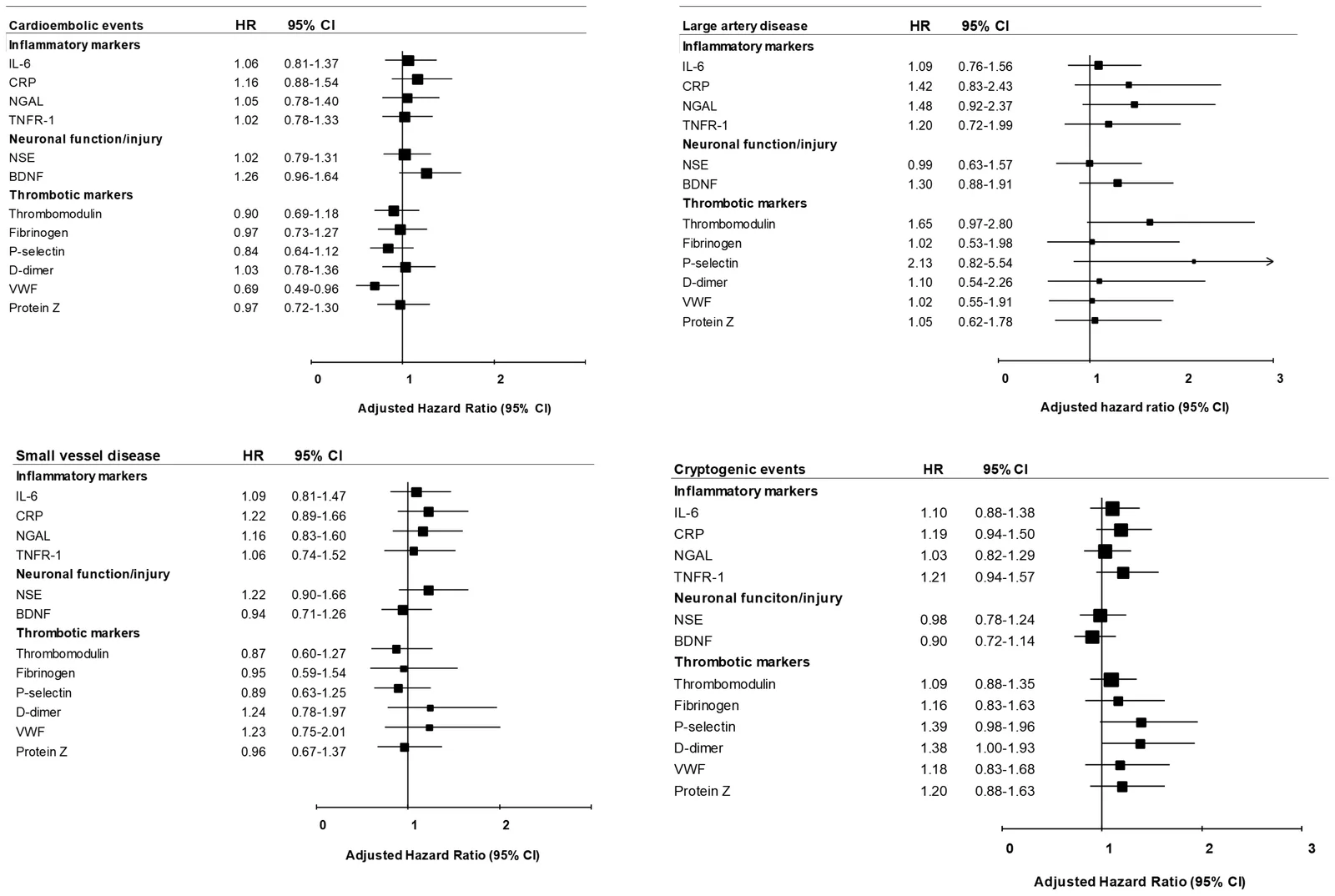
**Associations of individual biomarkers and long-term risks of recurrent stroke stratified by cause.** All analyses adjusted for age, sex, history of hypertension, diabetes, hyperlipidemia, atrial fibrillation, current smoking, chronic kidney disease, previous stroke, transient ischemic attack, peripheral arterial disease, and myocardial infarction. Hazard ratios (HRs) are presented as per SD(Ln) except for fibrinogen, VWF (von Willebrand Factor) antigen, and protein Z, which were given as SD. BDNF indicates brain-derived neurotrophic factor; CRP, C-reactive protein; IL-6, interleukin-6; NGAL, neutrophil gelatinase–associated lipocalin; NSE, neuron-specific enolase; and TNFR-1, tumor necrosis factor receptor-1.

Although no individual biomarker showed strong statistical significance with recurrent stroke during long-term follow-up, clustering of significantly elevated inflammatory biomarkers at baseline was associated with recurrent stroke (n/10-year risk: ≥3 elevated markers, 42/46.9% versus none, 114/21.3%; log-rank *P*<0.001; Figure 2; Figure S2). As expected, patients with clustering of inflammatory biomarkers were older and were more likely to have a history of vascular risk factors (Table S15), whereas no similar trend was seen for those with clustering of thrombotic markers (Table S16). In multivariate analyses adjusting for age, sex, and vascular risk factors, risks of recurrent stroke increased with increasing number of markers in the top quartile for inflammatory markers (1.21 [95% CI, 1.08–1.36]; *P*<0.001) but not for thrombotic markers (1.06 [95% CI, 0.91–1.23]; *P*=0.48). Moreover, clustering of elevated inflammatory markers was also associated with higher burden of recurrence (adjusted incidence rate ratio, 1.31 [95% CI, 1.15–1.50]; *P*<0.001). The association was strongest with inflammatory markers in patients with a known history of coronary artery disease (Table S17). Results were again consistent in sensitivity analyses excluding the first 90 days (inflammatory, 1.22 [95% CI, 1.07–1.39]; *P*=0.003; thrombotic, 1.03 [95% CI, 0.87–1.22]; *P*=0.71) when stratified by age (inflammatory/<70 years: 1.16 [95% CI, 0.91–1.47]; ≥70 years: 1.23 [95% CI, 1.08–1.41]) and in patients with TIA and minor stroke (inflammatory, 1.24 [95% CI, 1.07–1.44]; *P*=0.004; thrombotic, 1.04 [95% CI, 0.88–1.22]; *P*=0.69). Moreover, there was no convincing interaction with the status of neuronal or thrombotic markers although numerically clustering of inflammatory biomarkers tended to be more strongly associated with risks of recurrent stroke in those with lower burden of thrombotic markers compared with those with more marked increase and more strongly associated with risks of recurrent stroke in those with higher burden of neuronal markers compared with those with normal or mildly elevated neuronal markers (Table S18). A similar trend for the inflammatory biomarkers was also observed for the composite outcome of recurrent stroke, myocardial infarction, or sudden cardiac death (Table S19) although this was mainly driven by recurrent stroke, and there was no association with myocardial infarction (0.94 [95% CI, 0.74–1.20]; *P*=0.63) or myocardial infarction or sudden cardiac death (1.01 [95% CI, 0.83–1.23]; *P*=0.92). In the time-course analysis, clustering of inflammatory markers was also consistently associated with recurrent stroke up to 10 years (Figure 3; Table S20).

**Figure 2.**
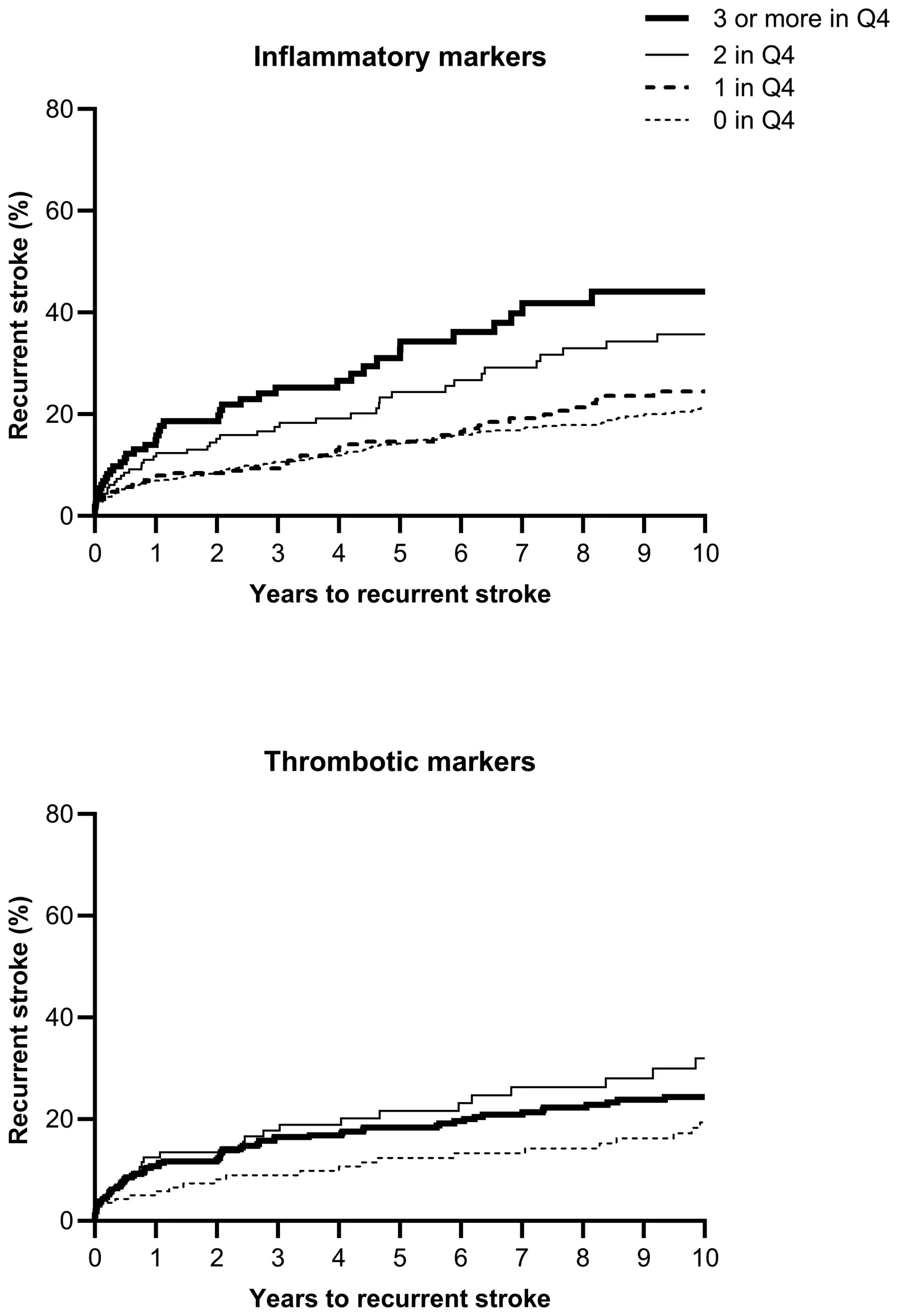
Ten-year risks of recurrent stroke stratified by numbers of biomarkers in the top quartile.

**Figure 3.**
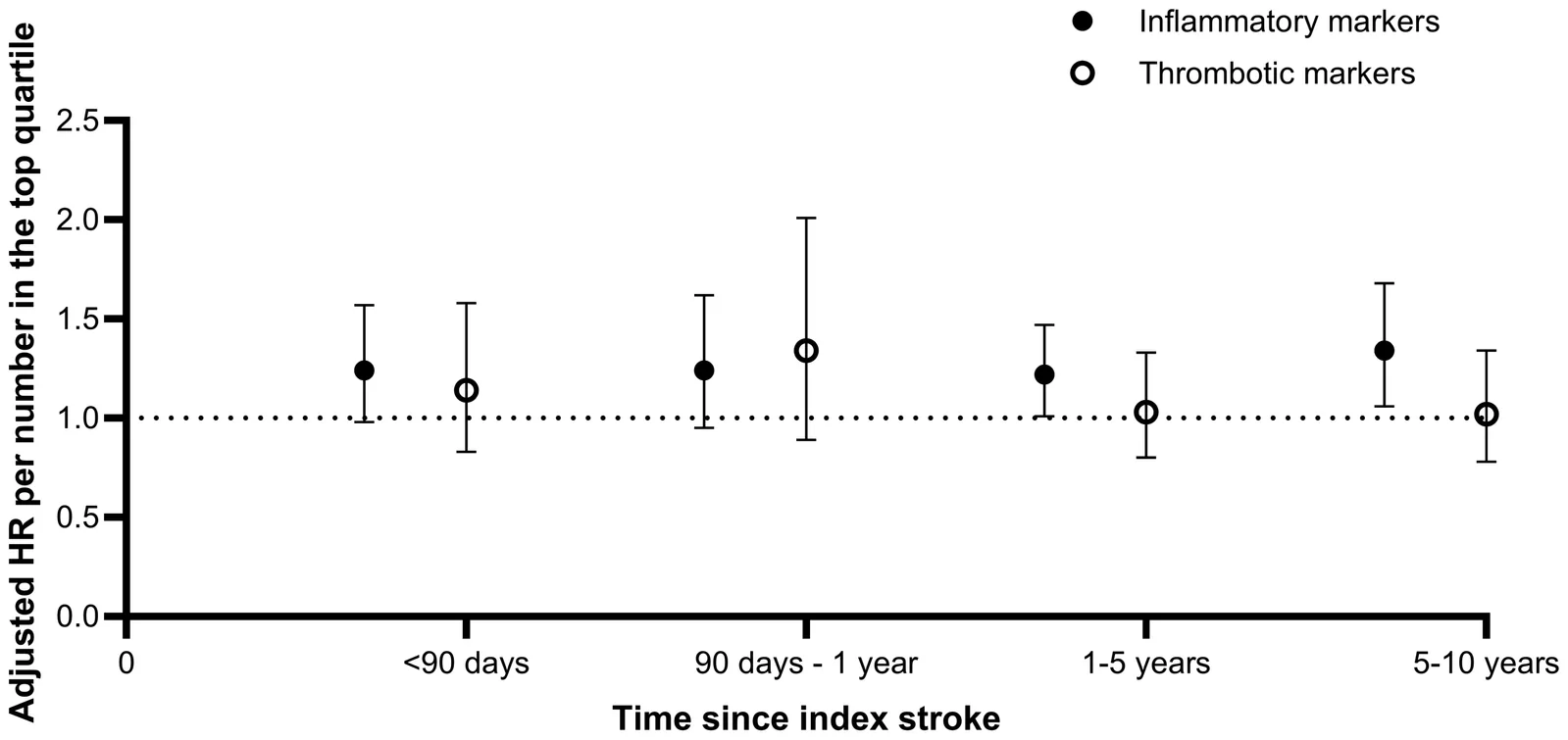
**Associations of clustering of biomarker type and long-term risks of recurrent stroke stratified by time since the index event.** All analyses adjusted for age, sex, history of hypertension, diabetes, hyperlipidemia, atrial fibrillation, current smoking, chronic kidney disease, previous stroke, transient ischemic attack, peripheral arterial disease, and myocardial infarction. HR indicates hazard ratio.

Analyses stratified by TOAST subtypes were presented in Table S21. Inflammatory markers tended to be more strongly associated with recurrent stroke in cardioembolic (adjusted hazard ratio, 1.41 [95% CI, 0.82–2.42]; *P*=0.21) and large artery disease (1.53 [95% CI, 0.66–3.55]; *P*=0.32), whereas thrombotic markers tended to be more strongly associated with recurrent stroke in large artery disease (4.65 [95% CI, 0.55–39.33]; *P*=0.16) and cryptogenic events (1.77 [95% CI, 0.78–4.03]; *P*=0.17). There was also some potential dose-response for inflammatory markers in cryptogenic events (per number of biomarkers in the top quartile, 1.24 [95% CI, 0.99–1.55]; *P*=0.06) and thrombotic markers for large vessel disease (2.26 [95% CI, 0.90–5.66]; *P*=0.08). It is also worth noting that there was significant heterogeneity in the cause of recurrent stroke versus the index event with only 80 of 135 (59.3%) and 84 of 157 (53.5%) having the same cause in those with higher levels of inflammatory or thrombotic markers, respectively.

## Discussion

In this large population-based study of TIA and stroke, we found limited predictive value for any individual thrombotic and neuronal cell damage biomarkers in assessing 10-year risk of recurrent stroke. However, individual inflammatory biomarkers showed some value, and more importantly, clustering of elevated levels in a panel of inflammatory markers was independently and more strongly associated with long-term risk and burden of recurrent stroke even after excluding the first 90 days and was persistent up to 10 years.

We have shown previously that IL-6 and CRP were predictors of early recurrent stroke,^30^ which is in line with other studies with short follow-up.^14^ In this study, we found that the predictive values of these individual markers in the longer term were attenuated. This is supported by a previous study with a mean follow-up of 2 years.^18^ Contrary to studies with a short follow-up,^20^ we also did not show convincing subtype-specific strong associations in the longer term. While this could be related to our statistical power in stratified analyses, it might also suggest that multiple inflammatory pathways might be involved in the association between inflammation and stroke in the longer term. Interestingly, we found that clustering of elevated levels across a panel of inflammatory markers was independently associated with long-term risk of recurrent stroke after the acute phase. In contrast, not all individual biomarkers retain statistical significance when considered in isolation. This finding is biologically plausible, as a panel capturing multiple inflammatory pathways may better reflect the overall burden of systemic inflammation than any single biomarker. Such an approach may, therefore, provide a more robust measure of risk. However, these observations require confirmation in independent cohorts, particularly to determine whether a multimarker panel offers incremental predictive value beyond established clinical risk factors. Our findings also support ongoing trials testing Colchicine, a broad-spectrum anti-inflammatory agent, in reducing recurrent stroke and other vascular events in patients with stroke and TIA.^32^

We also found some subgroups that are potentially interesting and should be considered in patient selection for future trials. For example, we found that clustering of inflammatory markers predicted long-term risk of recurrent stroke, particularly in those with a history of coronary artery disease, but the same effect was not seen for predicting long-term risk of myocardial infarction or sudden cardiac death. This is in line with the CONVINCE trial (Colchicine for Prevention of Vascular Inflammation in Non-Cardioembolic Stroke), which also showed Colchicine reduced risks of stroke, particularly in those with a known history of coronary artery disease, and Colchicine did not reduce risks of nonfatal myocardial or vascular death.^33^

Many of the other studied biomarkers were previously shown to be significantly associated with recurrent vascular events overall or death, but our study found that their predictive value for recurrent stroke might be limited, especially when adjusted for vascular risk factors. This is potentially plausible as these markers were likely to be associated with other underlying conditions, such as cancer or heart disease, or may have reflected an acute response to a short lifting trigger.^31^ Moreover, given recurrent stroke is much more common than myocardial infarction after stroke,^34^ and we also showed considerable heterogeneity in the cause of recurrent stroke versus their index stroke, these blood biomarkers might not be useful as a stratification tool to improve secondary prevention after stroke although studies to look at the prognostic values of these biomarkers in predicting nonstroke long-term vascular outcomes might still be interesting.

We found a weak association between D-dimer and recurrent stroke during long-term follow-up, and this signal was strongest in patients with cryptogenic events. Previous studies have also suggested that while D-dimer levels were not predictive of long-term recurrent stroke in cohorts that included all types of stroke,^35,36^ higher D-dimer at baseline was associated with increased risk of stroke in those with cryptogenic events,^37^ especially if the events were associated with patent foramen ovale.^38^ Higher D-dimer levels have also been linked to occult atrial fibrillation and cancer in patients with embolic cryptogenic events.^35^ However, most of these studies were relatively small with short follow-up, and different assays have been used in different studies, limiting the validation across studies. More studies using standardized methods are needed to determine if D-dimer has any potential to improve management decisions in secondary stroke prevention. Moreover, D-dimer has also been suggested to have a role in poststroke dementia and cognitive decline in the elderly,^39^ and future studies are warranted to further test this hypothesis.

The strengths of our study are its large sample size with unselected patients of TIA and stroke and 10-year follow-up, but there are also limitations. First, we only measured the biomarkers at baseline, so we were not able to identify a subgroup of patients who might have persistently increased levels during follow-up, who are potentially at higher risk of recurrent stroke. However, we previously showed in a small sample of 80 consecutive patients who had repeated testing at 1 year that all biomarkers but IL-6, NGAL, NSE, BDNF, and P-selectin were statistically significantly correlated between baseline and 1 year.^31^ Moreover, a recent study suggested that during the chronic phase in high-risk patients receiving statins, hsCRP was able to identify a subgroup of patients with residual inflammatory risk who were at increased future risks of cardiovascular events although the median follow-up was only around 5 years.^40^ Second, we were unable to measure all biomarkers in the entire cohort, and our statistical power was particularly limited for thrombotic markers although we did not see any signal apart from D-dimer. Moreover, a small number of patients who were on anticoagulation did not have the thrombotic markers assessed. Third, although we attempted to stratify the analyses by stroke cause, our numbers of end points for each stroke subtype were small, and we were not able to reliably exclude any possible weak subtype-specific associations. Fourth, for practical reasons, fasting was not required for blood sampling, which may have introduced additional variability to biomarker measurements. Fifth, although we adjusted for established clinical risk factors, residual confounding cannot be entirely ruled out. Sixth, other promising astroglial and neuronal injury biomarkers, such as glial fibrillary acidic protein, neurofilament light chain, and tau protein, were not measured. Last, our results may not be generalizable to non-White populations.

In conclusion, in a large population-based study of TIA and stroke, we found that inflammatory biomarkers were more predictive of long-term recurrent stroke than neuronal or thrombotic biomarkers, particularly when several inflammatory markers were raised. Predictive value was independent of other vascular risk factors and did not diminish with duration of follow-up, supporting trials of anti-inflammatory strategies for long-term secondary prevention. More research is still needed to determine if adding a panel of inflammatory biomarkers would enhance risk prediction in addition to established clinical risk factors.

## ARTICLE INFORMATION

### Acknowledgments

The authors are grateful to all the staff in the general practices that collaborated in the OXVASC (Oxford Vascular Study): Abingdon Surgery, Abingdon, United Kingdom; Malthouse Surgery, Abingdon; Marcham Road Family Health Centre, Abingdon; and The Health Centre, Berinsfield, United Kingdom; and key medical practice; Kidlington, Oxford, United Kingdom; East Oxford Health Centre, Oxford; and Church Street Practice, Wantage, United Kingdom. The authors are also grateful for our nonremunerated collaboration with Randox Laboratories. Written informed consent or assent from relatives was obtained from all participants. OXVASC was approved by the local research ethics committee (OREC A: 05/Q1604/70).

### Disclosures

None.

### Supplemental Material

Tables S1–S21

Figures S1–S2

STROBE Checklist

## Supplementary Material


