# Supplementary material for: Persistence of Association of Blood Biomarkers With Risk of Recurrent Stroke on Long-Term Follow-Up: Population-Based Study
Source: Stroke. 2026 Aug 3;57(10):3035–45. doi: 10.1161/STROKEAHA.126.056325 (PMC13619134; doi:10.1161/STROKEAHA.126.056325)

SUPPLEMENTAL MATERIALS

Persistence of association of blood biomarkers with risk of recurrent stroke on long-term follow-up: Population-based study

Linxin Li, Annette I Burgess, Julia Duerden, Katherine Onions, Helen C Segal, Peter M Rothwell

On behalf of the Oxford Vascular Study

Table S1 Intra- and inter-assay coefficients of variation of tested biomarkers

Biomarker	Intra-assay CV%	Inter-assay CV%
Inflammatory markers		
IL6 pg/ml (n=1207)	21	13.4
CRP mg/l (n=1207)	5.7	11.1
NGAL ng/ml (n=1207)	4.7	9.0
TNFR-1 ng/ml (n=1205)	7.8	14.1
Thrombotic markers		
TM ng/ml (n=1207)	10.1	14.1
Fibrinogen g/L (n=1030)	2.3	5.7
VWF iu/ml (n=1040)	4.6	4.6
P-selectin ng/ml (n=1072)	4.9	9.0
Protein Z ng/ml (n=1072)	4.9	14.8
D-dimer ng/ml (n=915)	4.3	4.3
Markers of neuronal function/injury		
NSE ng/ml (n=1207)	7.5	20.0
BDNF pg/ml (n=1207)	9.6	11.1

CV=coefficient of variation; IL-6=interleukin-6; CRP=C-reactive protein; NGAL=neutrophil gelatinase-associated lipocalin; TNF=tumour necrosis factor; NSE=neurone-specific enolase; BDNF=brain-derived neurotrophic factor; TM=thrombomodulin; VWF=von Willebrand factor antigen.

Table S2 Correlations between studied biomarkers (Spearman rank correlation, p-value)*

	CRP	NGAL	sTNFR-1	Thrombomodulin	Fibrinogen	VWF	P-Selectin	ProteinZ	D-Dimer	BDNF	NSE
IL6	0.48 (<0.0001)	0.42 (<0.0001)	0.34 (<0.0001)	0.16 (<0.0001)	0.31 (<0.0001)	0.29 (<0.0001)	0.09 (0.015)	-0.14 (0.0003)	0.26 (<0.0001)	0.14 (<0.0001)	0.15 (<0.0001)
CRP		0.34 (<0.0001)	0.39 (<0.0001)	0.12 (0.0004)	0.50 (<0.0001)	0.33 (<0.0001)	0.12 (0.0024)	-0.07 (0.086)	0.21 (<0.0001)	0.13 (0.0002)	0.13 (0.0002)
NGAL			0.53 (<0.0001)	0.31 (<0.0001)	0.24 (<0.0001)	0.27 (<0.0001)	0.19 (<0.0001)	-0.10 (0.016)	0.25 (<0.0001)	0.43 (<0.0001)	0.35 (<0.0001)
sTNFR-1				0.40 (<0.0001)	0.20 (<0.0001)	0.30 (<0.0001)	0.18 (<0.0001)	-0.10 (0.0083)	0.26 (<0.0001)	0.11 (0.0011)	0.21 (<0.0001)
Thrombomodulin					0.03 (0.45)	0.18 (<0.0001)	0.06 (0.11)	-0.04 (0.31)	0.17 (<0.0001)	0.09 (0.0057)	0.14 (<0.0001)
Fibrinogen						0.33 (<0.0001)	0.07 (0.077)	-0.05 (0.22)	0.15 (<0.0001)	0.07 (0.068)	0.03 (0.48)
VWF							0.01 (0.75)	-0.09 (0.022)	0.26 (<0.0001)	0.07 (0.074)	0.10 (0.0092)
P Selection								0.04 (0.34)	0.10 (0.0089)	0.09 (0.016)	0.10 (0.011)
Protein Z									-0.07 (0.068)	-0.05 (0.17)	-0.004 (0.92)
D-Dimer										0.03 (0.49)	0.11 (0.0045)
BDNF											0.12 (0.0003)
NSE											

*With the exception of Fibrinogen, vWF and PZ which were normally distributed, all biomarkers levels were log-transformed

IL-6=interleukin-6; CRP=C-reactive protein; NGAL=neutrophil gelatinase-associated lipocalin; TNFR=tumour necrosis factor receptor; NSE=neurone-specific enolase; BDNF=brain-derived neurotrophic factor; VWF=von Willebrand factor antigen.

Table S3 Associations of individual biomarkers with long-term risk of recurrent stroke stratified by previous history of coronary artery disease

	History of coronary artery disease		No history of coronary artery disease	
	**HR (95%CI)**	**p**	**HR (95%CI)**	**p**
**Inflammatory markers**†				
IL-6	1.32 (1.06-1.63)	0.013	1.02 (0.88-1.19)	0.78
CRP	1.36 (1.02-1.81)	0.036	1.16 (1.00-1.35)	0.053
NGAL	1.39 (1.01-1.92)	0.046	1.04 (0.90-1.21)	0.62
TNFR-1	1.39 (1.06-1.82)	0.019	1.03 (0.87-1.20)	0.77
**Neuronal function/injury**†				
NSE	1.03 (0.80-1.31)	0.84	1.06 (0.92-1.23)	0.40
BDNF	1.03 (0.80-1.33)	0.82	1.04 (0.90-1.20)	0.56
**Thrombotic markers**†				
Thrombomodulin	1.14 (0.85-1.53)	0.38	0.97 (0.82-1.13)	0.66
Fibrinogen	1.13 (0.86-1.49)	0.38	1.02 (0.84-1.23)	0.85
P-selectin	1.00 (0.72-1.39)	0.99	1.08 (0.91-1.28)	0.39
D-dimer	1.37 (0.97-1.94)	0.08	1.17 (0.97-1.43)	0.11
VWF	1.21 (0.88-1.64)	0.24	0.90 (0.74-1.09)	0.27
Protein Z	0.87 (0.63-1.22)	0.43	1.02 (0.86-1.22)	0.80

IL-6=interleukin-6; CRP=C-reactive protein; NGAL=neutrophil gelatinase-associated lipocalin; TNFR=tumour necrosis factor receptor; NSE=neurone-specific enolase; BDNF=brain-derived neurotrophic factor; VWF=von Willebrand factor antigen.
†Given per SD(Ln); fibrinogen, VWF and Protein Z were given as SD due to normal distribution of levels.
Model adjusted for age, sex, history of hypertension, diabetes, hyperlipidaemia, atrial fibrillation, current smoking, chronic kidney disease, previous stroke, transient ischaemic attack, peripheral arterial disease and myocardial infarction.

Table S4 Multivariate analyses of the associations of individual biomarkers with long-term risks of recurrent stroke stratified by follow-up period

	<90 days		90 days - 1 year		1-5 years		5-10 years	
	**HR (95%CI)**	**p**	**HR (95%CI)**	**p**	**HR (95%CI)**	**p**	**HR (95%CI)**	**p**
**Inflammatory markers**								
IL-6	1.11 (0.86-1.43)	0.43	1.21 (0.92-1.60)	0.18	1.07 (0.87-1.31)	0.52	1.23 (0.97-1.56)	0.09
CRP	1.56 (1.15-2.12)	0.004	1.06 (0.78-1.45)	0.71	1.33 (1.06-1.65)	0.01	1.08 (0.84-1.39)	0.53
NGAL	1.19 (0.89-1.60)	0.25	1.15 (0.81-1.63)	0.43	1.17 (0.93-1.47)	0.19	1.17 (0.91-1.49)	0.22
TNFR-1	1.16 (0.86-1.56)	0.33	1.06 (0.75-1.50)	0.75	1.12 (0.88-1.41)	0.36	1.25 (0.94-1.65)	0.13
**Neuronal function/injury**								
NSE	1.05 (0.81-1.37)	0.71	1.02 (0.75-1.39)	0.92	1.12 (0.91-1.37)	0.28	1.08 (0.86-1.35)	0.53
BDNF	1.11 (0.85-1.44)	0.45	1.12 (0.83-1.52)	0.46	1.06 (0.86-1.31)	0.57	1.03 (0.81-1.30)	0.82
**Thrombotic markers**								
Thrombomodulin	1.10 (0.86-1.42)	0.44	1.05 (0.75-1.47)	0.78	0.93 (0.73-1.17)	0.52	1.00 (0.78-1.28)	0.97
Fibrinogen	0.92 (0.69-1.24)	0.60	1.12 (0.82-1.52)	0.48	1.16 (0.90-1.49)	0.26	1.05 (0.75-1.46)	0.78
P-selectin	1.02 (0.76-1.35)	0.92	0.93 (0.67-1.29)	0.66	1.04 (0.81-1.34)	0.75	1.27 (0.92-1.77)	0.15
D-dimer	0.97 (0.68-1.40)	0.89	1.47 (0.98-2.21)	0.06	1.36 (1.02-1.81)	0.04	1.40 (1.00-1.95)	0.05
VWF	0.86 (0.60-1.23)	0.41	1.28 (0.89-1.85)	0.19	1.13 (0.87-1.47)	0.35	0.93 (0.66-1.31)	0.68
Protein Z	0.89 (0.64-1.23)	0.47	1.18 (0.82-1.71)	0.38	0.97 (0.75-1.25)	0.80	1.14 (0.85-1.53)	0.38

*Adjusted for age, sex, history of hypertension, diabetes, hyperlipidaemia, atrial fibrillation, current smoking, chronic kidney disease, previous stroke, transient ischaemic attack, peripheral arterial disease and myocardial infarction. Hazard ratios are presented as per SD(Ln) except for fibrinogen, VWF and Protein Z which were given as SD.
IL-6=interleukin-6; CRP=C-reactive protein; NGAL=neutrophil gelatinase-associated lipocalin; TNFR=tumour necrosis factor receptor; NSE=neurone-specific enolase; BDNF=brain-derived neurotrophic factor; VWF=von Willebrand factor antigen.

Table S5 Univariate and multivariate analyses of the associations of individual biomarkers with long-term risks of recurrent stroke in those with TIA or minor stroke

	**Unadjusted**		**Model 1**		**Model 2**	
	**HR (95%CI)**	**p**	**HR (95%CI)**	**p**	**HR (95%CI)**	**p**
**Inflammatory markers**†						
IL-6	1.17 (1.02-1.35)	0.03	1.09 (0.94-1.26)	0.26	1.07 (0.92-1.24)	0.38
CRP	1.18 (1.01-1.37)	0.03	1.13 (0.98-1.32)	0.10	1.13 (0.97-1.31)	0.13
NGAL	1.21 (1.05-1.40)	0.01	1.14 (0.98-1.32)	0.10	1.11 (0.95-1.30)	0.19
TNFR-1	1.33 (1.14-1.55)	0.0003	1.17 (1.00-1.38)	0.05	1.16 (0.98-1.37)	0.08
**Neuronal function/injury**†						
NSE	0.98 (0.85-1.13)	0.80	0.98 (0.85-1.14)	0.81	1.00 (0.86-1.16)	0.98
BDNF	0.94 (0.82-1.09)	0.41	0.96 (0.83-1.11)	0.55	0.97 (0.84-1.13)	0.70
**Thrombotic markers**†						
Thrombomodulin	1.08 (0.96-1.22)	0.20	1.03 (0.89-1.20)	0.65	1.06 (0.91-1.22)	0.47
Fibrinogen	1.05 (0.86-1.29)	0.62	0.97 (0.78-1.20)	0.75	0.96 (0.78-1.18)	0.72
P-selectin	1.10 (0.93-1.31)	0.26	1.10 (0.93-1.30)	0.27	1.07 (0.90-1.27)	0.44
D-dimer	1.45 (1.19-1.76)	0.0002	1.31 (1.07-1.61)	0.01	1.28 (1.04-1.58)	0.02
VWF	1.18 (0.97-1.44)	0.10	1.03 (0.83-1.29)	0.77	0.97 (0.78-1.21)	0.80
Protein Z	0.99 (0.82-1.19)	0.90	1.05 (0.88-1.27)	0.59	1.06 (0.88-1.27)	0.56

IL-6=interleukin-6; CRP=C-reactive protein; NGAL=neutrophil gelatinase-associated lipocalin; TNFR=tumour necrosis factor receptor; NSE=neurone-specific enolase; BDNF=brain-derived neurotrophic factor; VWF=von Willebrand factor antigen.
†Given per SD(Ln); fibrinogen, VWF and Protein Z were given as SD due to normal distribution of levels.
Model 1: adjusted for age and sex.
Model 2: adjusted for age, sex, history of hypertension, diabetes, hyperlipidaemia, atrial fibrillation, current smoking, chronic kidney disease, previous stroke, transient ischaemic attack, peripheral arterial disease and myocardial infarction.

Table S6 Univariate and multivariate analyses of the associations of individual biomarkers with long-term risks of recurrent stroke in those with TIA or minor stroke and excluding the first 90 days

	**Unadjusted**		**Model 1**		**Model 2**	
	**HR (95%CI)**	**p**	**HR (95%CI)**	**p**	**HR (95%CI)**	**p**
**Inflammatory markers**†						
IL-6	1.18 (1.01-1.37)	0.04	1.08 (0.92-1.27)	0.35	1.05 (0.89-1.24)	0.57
CRP	1.13 (0.95-1.33)	0.16	1.08 (0.91-1.28)	0.38	1.06 (0.89-1.26)	0.53
NGAL	1.22 (1.04-1.43)	0.02	1.14 (0.96-1.35)	0.13	1.10 (0.92-1.30)	0.30
TNFR-1	1.36 (1.14-1.62)	0.001	1.18 (0.99-1.42)	0.07	1.16 (0.97-1.40)	0.12
**Neuronal function/injury**†						
NSE	0.95 (0.81-1.12)	0.56	0.96 (0.81-1.13)	0.60	0.97 (0.82-1.15)	0.71
BDNF	0.92 (0.78-1.08)	0.31	0.94 (0.80-1.11)	0.45	0.95 (0.81-1.12)	0.52
**Thrombotic markers**†						
Thrombomodulin	1.06 (0.93-1.22)	0.40	1.00 (0.84-1.19)	0.99	1.01 (0.85-1.21)	0.88
Fibrinogen	1.05 (0.84-1.32)	0.66	0.95 (0.75-1.20)	0.66	0.97 (0.77-1.22)	0.78
P-selectin	1.10 (0.90-1.35)	0.33	1.11 (0.91-1.34)	0.31	1.05 (0.86-1.29)	0.60
D-dimer	1.57 (1.27-1.94)	<0.0001	1.40 (1.12-1.75)	0.003	1.39 (1.10-1.76)	0.005
VWF	1.22 (0.98-1.51)	0.07	1.04 (0.82-1.33)	0.73	1.00 (0.77-1.28)	0.97
Protein Z	1.00 (0.81-1.22)	0.98	1.08 (0.88-1.32)	0.49	1.08 (0.88-1.33)	0.44

IL-6=interleukin-6; CRP=C-reactive protein; NGAL=neutrophil gelatinase-associated lipocalin; TNFR=tumour necrosis factor receptor; NSE=neurone-specific enolase; BDNF=brain-derived neurotrophic factor; VWF=von Willebrand factor antigen.
†Given per SD(Ln); fibrinogen, VWF and Protein Z were given as SD due to normal distribution of levels.
Model 1: adjusted for age and sex.
Model 2: adjusted for age, sex, history of hypertension, diabetes, hyperlipidaemia, atrial fibrillation, current smoking, chronic kidney disease, previous stroke, transient ischaemic attack, peripheral arterial disease and myocardial infarction.

Table S7 Univariate and multivariate analyses of the associations of individual biomarkers with long-term risks of recurrent stroke in those presented with stroke

	Unadjusted		Model 1		Model 2	
	**HR (95%CI)**	**p**	**HR (95%CI)**	**p**	**HR (95%CI)**	**p**
**Inflammatory markers**†						
IL-6	1.20 (1.05-1.37)	0.009	1.11 (0.96-1.28)	0.17	1.09 (0.94-1.26)	0.27
CRP	1.26 (1.09-1.46)	0.002	1.20 (1.04-1.40)	0.02	1.21 (1.04-1.41)	0.02
NGAL	1.19 (1.02-1.38)	0.03	1.13 (0.97-1.31)	0.13	1.09 (0.93-1.28)	0.29
TNFR-1	1.32 (1.13-1.54)	0.0004	1.19 (1.01-1.40)	0.04	1.17 (0.99-1.38)	0.07
**Neuronal function/injury**†						
NSE	1.08 (0.94-1.24)	0.28	1.08 (0.94-1.24)	0.29	1.07 (0.93-1.23)	0.36
BDNF	1.03 (0.90-1.18)	0.66	1.02 (0.89-1.16)	0.79	1.01 (0.88-1.16)	0.85
**Thrombotic markers**†						
Thrombomodulin	1.08 (0.94-1.23)	0.28	1.03 (0.89-1.19)	0.67	1.04 (0.89-1.21)	0.62
Fibrinogen	1.08 (0.91-1.28)	0.39	1.02 (0.86-1.22)	0.82	1.04 (0.87-1.24)	0.68
P-selectin	0.98 (0.81-1.19)	0.86	0.97 (0.80-1.17)	0.74	0.95 (0.79-1.15)	0.61
D-dimer	1.29 (1.06-1.56)	0.01	1.20 (0.98-1.48)	0.08	1.12 (0.91-1.38)	0.30
VWF	1.04 (0.87-1.24)	0.70	0.96 (0.79-1.16)	0.65	0.92 (0.76-1.12)	0.40
Protein Z	0.90 (0.76-1.07)	0.25	0.94 (0.79-1.12)	0.46	0.95 (0.79-1.14)	0.58

IL-6=interleukin-6; CRP=C-reactive protein; NGAL=neutrophil gelatinase-associated lipocalin; TNFR=tumour necrosis factor receptor; NSE=neurone-specific enolase; BDNF=brain-derived neurotrophic factor; VWF=von Willebrand factor antigen.
†Given per SD(Ln); fibrinogen, VWF and Protein Z were given as SD due to normal distribution of levels.
Model 1: adjusted for age and sex.
Model 2: adjusted for age, sex, history of hypertension, diabetes, hyperlipidaemia, atrial fibrillation, current smoking, chronic kidney disease, previous stroke, transient ischaemic attack, peripheral arterial disease and myocardial infarction.

Table S8 Univariate and multivariate analyses of the associations of individual biomarkers with long-term risks of recurrent stroke in those presenting with stroke and excluding the first 90 days

	Unadjusted		Model 1		Model 2	
	**HR (95%CI)**	**p**	**HR (95%CI)**	**p**	**HR (95%CI)**	**p**
**Inflammatory markers†**						
IL-6	1.24 (1.06-1.44)	0.007	1.13 (0.96-1.33)	0.14	1.13 (0.95-1.33)	0.16
CRP	1.19 (1.01-1.40)	0.04	1.13 (0.95-1.34)	0.16	1.14 (0.96-1.36)	0.13
NGAL	1.19 (1.00-1.41)	0.05	1.12 (0.94-1.34)	0.19	1.09 (0.91-1.31)	0.35
TNFR-1	1.37 (1.14-1.65)	0.001	1.20 (0.99-1.45)	0.06	1.18 (0.97-1.44)	0.11
**Neuronal function/injury†**						
NSE	1.08 (0.92-1.27)	0.34	1.09 (0.93-1.28)	0.30	1.09 (0.93-1.29)	0.30
BDNF	1.01 (0.87-1.18)	0.87	1.00 (0.86-1.17)	0.99	1.01 (0.86-1.18)	0.89
**Thrombotic markers†**						
Thrombomodulin	1.07 (0.92-1.25)	0.39	1.02 (0.86-1.21)	0.83	1.02 (0.85-1.21)	0.87
Fibrinogen	1.04 (0.85-1.28)	0.68	0.96 (0.78-1.18)	0.70	1.02 (0.82-1.26)	0.88
P-selectin	1.08 (0.85-1.38)	0.51	1.07 (0.84-1.36)	0.58	1.05 (0.82-1.34)	0.71
D-dimer	1.43 (1.14-1.79)	0.002	1.31 (1.03-1.67)	0.03	1.21 (0.95-1.54)	0.13
VWF	1.11 (0.91-1.36)	0.30	1.02 (0.82-1.27)	0.87	0.99 (0.80-1.23)	0.93
Protein Z	0.90 (0.74-1.10)	0.29	0.94 (0.76-1.15)	0.52	0.93 (0.75-1.14)	0.47

IL-6=interleukin-6; CRP=C-reactive protein; NGAL=neutrophil gelatinase-associated lipocalin; TNFR=tumour necrosis factor receptor; NSE=neurone-specific enolase; BDNF=brain-derived neurotrophic factor; VWF=von Willebrand factor antigen.
†Given per SD(Ln); fibrinogen, VWF and Protein Z were given as SD due to normal distribution of levels.
Model 1: adjusted for age and sex.
Model 2: adjusted for age, sex, history of hypertension, diabetes, hyperlipidaemia, atrial fibrillation, current smoking, chronic kidney disease, previous stroke, transient ischaemic attack, peripheral arterial disease and myocardial infarction.

Table S9 Multivariate analyses of the associations of individual biomarkers with long-term risks of recurrent stroke using per quartile and top vs. bottom quartile

	**Fully adjusted model:** HR per quartile (95%CI)	**p**	**HR Q4 vs. Q1 (95%CI)**	**P**
**All follow-up:**				
**Inflammatory markers**				
IL-6	1.13 (1.00-1.27)	0.04	1.30 (0.91-1.86)	0.15
CRP	1.16 (1.03-1.30)	0.01	1.67 (1.16-2.41)	0.006
NGAL	1.12 (1.00-1.26)	0.06	1.40 (0.98-2.00)	0.07
TNFR-1	1.05 (0.93-1.18)	0.43	1.26 (0.88-1.82)	0.21
**Neuronal function/injury**				
NSE	1.08 (0.97-1.21)	0.17	1.26 (0.90-1.78)	0.19
BDNF	1.06 (0.95-1.18)	0.29	1.15 (0.81-1.61)	0.44
**Thrombotic markers**				
Thrombomodulin	1.01 (0.90-1.13)	0.89	1.09 (0.77-1.54)	0.64
Fibrinogen	1.09 (0.95-1.25)	0.25	1.29 (0.83-2.01)	0.26
P-selectin	1.11 (0.98-1.26)	0.11	1.32 (0.89-1.96)	0.17
D-dimer	1.17 (1.00-1.37)	0.05	1.37 (0.87-2.17)	0.18
VWF	1.02 (0.88-1.17)	0.82	1.07 (0.68-1.68)	0.78
Protein Z	0.99 (0.87-1.14)	0.91	0.90 (0.58-1.40)	0.63
**Excluding the first 90 days:**				
**Inflammatory markers**				
IL-6	1.13 (0.99-1.29)	0.08	1.29 (0.85-1.93)	0.23
CRP	1.11 (0.97-1.26)	0.12	1.46 (0.97-2.20)	0.07
NGAL	1.12 (0.98-1.28)	0.09	1.41 (0.94-2.10)	0.10
TNF receptor-1	1.05 (0.92-1.20)	0.48	1.25 (0.83-1.89)	0.29
**Neuronal function/injury**				
NSE	1.08 (0.95-1.23)	0.23	1.24 (0.84-1.83)	0.29
BDNF	1.04 (0.92-1.18)	0.54	1.11 (0.74-1.64)	0.62
**Thrombotic markers**				
Thrombomodulin	0.99 (0.87-1.13)	0.91	1.01 (0.68-1.51)	0.95
Fibrinogen	1.07 (0.91-1.25)	0.41	1.32 (0.80-2.19)	0.28
P-selectin	1.14 (0.98-1.33)	0.08	1.35 (0.86-2.13)	0.20
D-dimer	1.24 (1.03-1.48)	0.02	1.49 (0.89-2.47)	0.13
VWF	1.05 (0.89-1.24)	0.56	1.16 (0.69-1.95)	0.57
Protein Z	1.02 (0.88-1.20)	0.77	0.96 (0.58-1.59)	0.89

IL-6=interleukin-6; CRP=C-reactive protein; NGAL=neutrophil gelatinase-associated lipocalin; TNFR=tumour necrosis factor receptor; NSE=neurone-specific enolase; BDNF=brain-derived neurotrophic factor; VWF=von Willebrand factor antigen.

Table S10 Multivariate analyses of the associations of individual biomarkers with long-term risk of recurrent stroke taking into account delays from onset to sampling

	**All events***		**Further adjusting for delay to sampling***		**Excluding measurements taken in the first 24 hours***	
	**HR (95%CI)**	**p**	**HR (95%CI)**	**p**	**HR (95%CI)**	**p**
**Inflammatory markers**†						
IL-6	1.12 (0.99-1.26)	0.07	1.11 (0.99-1.26)	0.08	1.15 (1.01-1.31)	0.03
CRP	1.20 (1.05-1.37)	0.006	1.19 (1.05-1.36)	0.008	1.26 (1.09-1.46)	0.002
NGAL	1.11 (0.97-1.27)	0.13	1.11 (0.97-1.27)	0.12	1.13 (0.97-1.31)	0.12
TNFR-1	1.12 (0.97-1.29)	0.11	1.12 (0.98-1.29)	0.11	1.16 (0.99-1.35)	0.07
**Neuronal function/injury**†						
NSE	1.06 (0.93-1.20)	0.38	1.05 (0.93-1.19)	0.41	1.08 (0.95-1.24)	0.26
BDNF	1.05 (0.92-1.18)	0.48	1.05 (0.93-1.19)	0.44	1.02 (0.89-1.17)	0.78
**Thrombotic markers**†						
Thrombomodulin	1.01 (0.88-1.15)	0.94	1.00 (0.88-1.15)	0.97	0.98 (0.85-1.14)	0.81
Fibrinogen	1.05 (0.90-1.23)	0.54	1.05 (0.89-1.22)	0.59	1.06 (0.90-1.26)	0.48
P-selectin	1.06 (0.91-1.23)	0.46	1.06 (0.91-1.24)	0.44	1.05 (0.89-1.24)	0.57
D-dimer	1.21 (1.02-1.44)	0.03	1.21 (1.02-1.44)	0.03	1.28 (1.06-1.55)	0.01
VWF	0.98 (0.83-1.16)	0.83	0.98 (0.83-1.16)	0.83	0.97 (0.80-1.16)	0.72
Protein Z	1.00 (0.86-1.17)	0.99	1.00 (0.86-1.17)	0.96	1.03 (0.87-1.22)	0.72

IL-6=interleukin-6; CRP=C-reactive protein; NGAL=neutrophil gelatinase-associated lipocalin; TNFR=tumour necrosis factor receptor; NSE=neurone-specific enolase; BDNF=brain-derived neurotrophic factor; VWF=von Willebrand factor antigen.
†Given per SD(Ln); fibrinogen, VWF and Protein Z were given as SD due to normal distribution of levels.
*Adjusted for age, sex, history of hypertension, diabetes, hyperlipidaemia, atrial fibrillation, current smoking, chronic kidney disease, previous stroke, transient ischaemic attack, peripheral arterial disease and myocardial infarction.

Table S11 Multivariate analyses of the associations of individual biomarkers with long-term risk of recurrent stroke or myocardial infarction and sudden cardiac death

	All events*		TIA/minor stroke*		Excluding the first 90 days*	
	**HR (95%CI)**	**p**	**HR (95%CI)**	**p**	**HR (95%CI)**	**p**
**Inflammatory markers**†						
IL-6	1.12 (1.01-1.25)	0.03	1.09 (0.96-1.24)	0.17	1.13 (1.00-1.27)	0.047
CRP	1.15 (1.03-1.30)	0.02	1.11 (0.97-1.27)	0.14	1.10 (0.97-1.25)	0.15
NGAL	1.11 (0.99-1.26)	0.08	1.14 (1.00-1.31)	0.06	1.10 (0.96-1.25)	0.18
TNFR-1	1.07 (0.95-1.21)	0.28	1.11 (0.96-1.28)	0.18	1.06 (0.92-1.21)	0.45
**Neuronal function/injury**†						
NSE	1.10 (0.99-1.22)	0.08	1.08 (0.96-1.23)	0.21	1.09 (0.97-1.23)	0.15
BDNF	1.05 (0.94-1.17)	0.41	1.02 (0.89-1.16)	0.82	1.04 (0.92-1.18)	0.52
**Thrombotic markers**†						
Thrombomodulin	0.98 (0.87-1.10)	0.71	1.02 (0.89-1.16)	0.82	0.95 (0.82-1.09)	0.44
Fibrinogen	1.06 (0.93-1.21)	0.39	1.02 (0.86-1.21)	0.79	1.04 (0.89-1.21)	0.62
P-selectin	1.06 (0.92-1.21)	0.44	1.06 (0.91-1.23)	0.46	1.06 (0.91-1.24)	0.44
D-dimer	1.16 (1.00-1.34)	0.06	1.24 (1.04-1.47)	0.02	1.19 (1.01-1.41)	0.03
VWF	0.99 (0.86-1.15)	0.92	0.99 (0.82-1.20)	0.95	1.00 (0.85-1.18)	0.97
Protein Z	0.97 (0.85-1.11)	0.64	0.99 (0.85-1.16)	0.91	0.99 (0.85-1.15)	0.90

IL-6=interleukin-6; CRP=C-reactive protein; NGAL=neutrophil gelatinase-associated lipocalin; TNFR=tumour necrosis factor receptor; NSE=neurone-specific enolase; BDNF=brain-derived neurotrophic factor; VWF=von Willebrand factor antigen.
†Given per SD(Ln); fibrinogen, VWF and Protein Z were given as SD due to normal distribution of levels.
*Adjusted for age, sex, history of hypertension, diabetes, hyperlipidaemia, atrial fibrillation, current smoking, chronic kidney disease, previous stroke, transient ischaemic attack, peripheral arterial disease and myocardial infarction.

Table S12 Multivariate analyses of the associations of individual biomarkers with long-term risk of myocardial infarction and sudden cardiac death (n=111)

	All follow-up:						Excluding first 90d:		Known history of coronary artery disease:	
	**Unadjusted**		**Age/sex adjusted**		**Multivariate***		**Multivariate***		**Multivariate***	
	**HR (95%CI)**	**p**	**HR (95%CI)**	**p**	**HR (95%CI)**	**p**	**HR (95%CI)**	**p**	**HR (95%CI)**	**p**
**Inflammatory markers†**										
IL-6	1.30 (1.09-1.55)	0.003	1.20 (0.99-1.44)	0.06	1.11 (0.91-1.35)	0.31	1.09 (0.89-1.34)	0.42	0.81 (0.57-1.15)	0.24
CRP	1.12 (0.91-1.39)	0.27	1.09 (0.88-1.35)	0.43	1.02 (0.82-1.27)	0.86	1.03 (0.82-1.30)	0.80	0.93 (0.64-1.34)	0.68
NGAL	1.36 (1.09-1.69)	0.006	1.21 (0.97-1.52)	0.10	1.10 (0.87-1.40)	0.42	1.09 (0.85-1.39)	0.50	0.96 (0.67-1.39)	0.84
TNFR-1	1.30 (1.05-1.62)	0.02	1.07 (0.85-1.34)	0.56	0.89 (0.70-1.13)	0.34	0.89 (0.70-1.15)	0.37	0.63 (0.41-0.95)	0.03
**Neuronal function/injury†**										
NSE	1.19 (0.99-1.45)	0.07	1.18 (0.97-1.43)	0.09	1.18 (0.97-1.43)	0.10	1.12 (0.91-1.37)	0.30	1.04 (0.76-1.42)	0.79
BDNF	1.02 (0.83-1.25)	0.85	1.03 (0.84-1.27)	0.75	0.99 (0.81-1.22)	0.93	1.00 (0.81-1.24)	0.98	0.92 (0.67-1.27)	0.62
**Thrombotic markers†**										
Thrombomodulin	1.08 (0.90-1.29)	0.40	0.96 (0.77-1.21)	0.73	0.89 (0.70-1.14)	0.36	0.89 (0.69-1.14)	0.36	0.64 (0.41-0.99)	0.045
Fibrinogen	1.10 (0.87-1.39)	0.41	1.02 (0.80-1.30)	0.88	1.03 (0.82-1.31)	0.78	0.99 (0.77-1.27)	0.95	1.01 (0.73-1.41)	0.95
P-selectin	1.18 (0.91-1.52)	0.21	1.17 (0.91-1.50)	0.21	1.06 (0.82-1.35)	0.67	1.05 (0.82-1.35)	0.71	0.96 (0.64-1.44)	0.85
D-dimer	1.16 (0.91-1.47)	0.24	0.99 (0.77-1.26)	0.90	0.92 (0.72-1.19)	0.54	0.90 (0.70-1.17)	0.43	0.98 (0.66-1.44)	0.91
VWF	1.18 (0.94-1.49)	0.16	1.02 (0.79-1.33)	0.86	0.98 (0.74-1.29)	0.88	0.87 (0.64-1.17)	0.34	1.07 (0.70-1.65)	0.75
Protein Z	0.81 (0.64-1.02)	0.08	0.87 (0.68-1.10)	0.25	0.88 (0.69-1.12)	0.30	0.88 (0.69-1.13)	0.33	0.97 (0.66-1.45)	0.90

IL-6=interleukin-6; CRP=C-reactive protein; NGAL=neutrophil gelatinase-associated lipocalin; TNFR=tumour necrosis factor receptor; NSE=neurone-specific enolase; BDNF=brain-derived neurotrophic factor; VWF=von Willebrand factor antigen.

†Given per SD(Ln); fibrinogen, vWF and Protein Z were given as SD due to normal distribution of levels.

*Adjusted for age, sex, history of hypertension, diabetes, hyperlipidaemia, atrial fibrillation, current smoking, chronic kidney disease, previous stroke, transient ischaemic attack, peripheral arterial disease and myocardial infarction.

Table S13 Baseline biomarker levels (median/IQR) by TOAST subtypes

	**Cardioembolic**	**Large artery**	**Small vessel**	**Cryptogenic**
**Age (mean/SD)**	78.2/12.1	73.9/10.2	70.6/12.3	71.3/13.0
**Inflammatory markers**				
IL-6, pg/ml *	4.48 (1.61-11.35)	2.07 (1.20-7.26)	1.80 (0.86-4.03)	1.52 (0.77-3.35)
CRP, mg/l *	8.41 (2.26-12.00)	4.24 (1.89-11.08)	3.12 (1.35-10.05)	2.39 (1.09-6.28)
NGAL, ng/ml *	943.00 (653.50-1229.00)	870.00 (643.75-1154.50)	765.00 (535.25-1059.75)	698.00 (495.00-1011.00)
TNFR-1, ng/ml *	1.02 (0.70-1.50)	1.02 (0.77-1.33)	0.78 (0.58-1.09)	0.80 (0.58-1.11)
**Neuronal function/injury**				
NSE, ng/ml *	8.27 (5.41-12.75)	7.82 (4.67-12.43)	6.70 (4.52-9.70)	6.27 (4.15-10.00)
BDNF, pg/ml *	1055.50 (596.00-1762.25)	980.00 (54.00-1488.75)	918.00 (456.00-1499.50)	756.00 (471.25-1261.50)
**Thrombotic markers**				
Thrombomodulin, ng/ml *	1.57 (1.30-2.02)	1.77 (1.45-2.16)	1.69 (1.41-2.10)	1.65 (1.35-2.07)
Fibrinogen, g/l	443.00 (363.75-512.25)	412.50 (354.00-486.50)	402.00 (342.00-482.00)	386.00 (328.75-465.75)
P-selectin, ng/ml *	41.00 (25.00-59.25)	40.00 (19.00-52.00)	32.50 (18.00-51.25)	31.50 (19.00-43.00)
D-dimer, ug/l	679.50 (343.25-1343.00)	591.00 (293.00-928.00)	521.00 (286.00-1032.00)	432.00 (266.50-854.00)
VWF, IU/ml *	206.00 (154.50-247.00)	167.00 (135.00-206.50)	145.00 (117.50-186.25)	155.50 (115.00-198.50)
Protein Z, ng/ml	1.64 (1.25-2.12)	1.63 (1.14-2.06)	1.77 (1.30-2.18)	1.86 (1.40-2.24)

*significant difference between subtypes after adjusting for age and sex with log transformed values of studied biomarkers. VWF and Protein Z were not transformed as they were normally distributed. IL-6=interleukin-6; CRP=C-reactive protein; NGAL=neutrophil gelatinase-associated lipocalin; TNFR=tumour necrosis factor receptor; NSE=neurone-specific enolase; BDNF=brain-derived neurotrophic factor; VWF=von Willebrand factor antigen.

Table S14 Multivariate analyses* of the associations of individual biomarkers with long-term risks of recurrent stroke in those with TIA or minor stroke and excluding the first 90 days with stratification by stroke aetiology

	Cardioembolic events		Large artery disease		Small vessel disease		Cryptogenic events	
	**HR (95%CI)**	**p**	**HR (95%CI)**	**p**	**HR (95%CI)**	**p**	**HR (95%CI)**	**p**
**Inflammatory markers**								
IL-6	0.88 (0.57-1.34)	0.55	1.21 (0.73-2.01)	0.47	0.89 (0.57-1.38)	0.60	1.07 (0.79-1.45)	0.65
CRP	0.93 (0.60-1.44)	0.76	1.31 (0.58-2.94)	0.52	1.06 (0.71-1.59)	0.76	1.07 (0.81-1.41)	0.64
NGAL	0.90 (0.55-1.46)	0.66	1.65 (0.81-3.32)	0.17	1.06 (0.72-1.55)	0.76	1.01 (0.77-1.32)	0.96
TNFR-1	1.10 (0.70-1.73)	0.68	1.00 (0.47-2.13)	1.00	1.14 (0.74-1.76)	0.56	1.25 (0.92-1.70)	0.16
**Neuronal function/injury**								
NSE	0.88 (0.58-1.34)	0.55	1.13 (0.60-2.11)	0.70	1.14 (0.74-1.77)	0.56	0.94 (0.71-1.25)	0.67
BDNF	0.98 (0.63-1.51)	0.92	1.50 (0.84-2.69)	0.17	0.73 (0.52-1.03)	0.07	0.98 (0.73-1.31)	0.88
**Thrombotic markers**								
Thrombomodulin	0.93 (0.58-1.49)	0.77	0.97 (0.39-2.41)	0.95	1.01 (0.63-1.62)	0.97	1.02 (0.78-1.35)	0.87
Fibrinogen	1.05 (0.70-1.59)	0.81	31.53 (1.33-745.38)	0.03	0.68 (0.37-1.26)	0.22	0.84 (0.50-1.41)	0.51
P-selectin	0.78 (0.50-1.23)	0.29	7.58 (0.71-81.41)	0.09	0.81 (0.51-1.28)	0.36	1.41 (0.93-2.15)	0.11
D-dimer	1.04 (0.64-1.70)	0.86	2.14 (0.58-7.92)	0.25	1.15 (0.68-1.97)	0.60	1.45 (0.99-2.13)	0.06
VWF	0.96 (0.57-1.64)	0.89	0.95 (0.35-2.57)	0.92	1.46 (0.64-3.31)	0.37	1.04 (0.61-1.77)	0.89
Protein Z	0.98 (0.60-1.61)	0.94	1.18 (0.38-3.70)	0.77	1.08 (0.69-1.68)	0.75	1.31 (0.90-1.89)	0.16

*Adjusted for age, sex, history of hypertension, diabetes, hyperlipidaemia, atrial fibrillation, current smoking, chronic kidney disease, previous stroke, transient ischaemic attack, peripheral arterial disease and myocardial infarction.
Hazard ratios are presented as per SD(Ln) except for fibrinogen, VWF and Protein Z which were given as SD.
IL-6=interleukin-6; CRP=C-reactive protein; NGAL=neutrophil gelatinase-associated lipocalin; TNFR=tumour necrosis factor receptor; NSE=neurone-specific enolase; BDNF=brain-derived neurotrophic factor; VWF=von Willebrand factor antigen.

Table S15 Baseline characteristics of patients with different inflammatory profiles

	No marker in the top quartile (n=610)	One marker in the top quartile (n=231)	Two markers in the top quartile (n=187)	Three or more markers in the top quartile (n=173)	p for trend
**Age (mean/SD)**	70.1/13.7	73.1/12.9	77.0/10.9	80.6/9.3	<0.001
**Sex**	290 (47.5)	119 (51.5)	82 (43.9)	81 (46.8)	0.63
**Hypertension**	331 (54.3)	131 (56.7)	130 (69.5)	122 (70.5)	<0.001
**Diabetes**	61 (10.0)	27 (11.7)	25 (13.4)	27 (15.6)	0.03
**Hyperlipidaemia**	201 (33.0)	77 (33.3)	58 (31.0)	49 (28.3)	0.26
**Atrial fibrillation**	73 (12.0)	37 (16.0)	45 (24.1)	56 (32.4)	<0.001
**Current smoking**	94 (15.4)	41 (17.7)	30 (16.0)	21 (12.1)	0.47
**Chronic kidney disease**	272 (44.6)	127 (55.0)	117 (62.6)	121 (69.9)	<0.001
**Previous MI**	42 (6.9)	26 (11.3)	23 (12.3)	27 (15.6)	<0.001
**Previous PVD**	30 (4.9)	23 (10.0)	18 (9.6)	26 (15.0)	<0.001
**Previous stroke**	69 (11.3)	25 (10.8)	27 (14.4)	34 (19.7)	0.005
**Previous TIA**	90 (14.8)	29 (12.6)	26 (13.9)	16 (9.2)	0.10

Numbers are reported as n (%) unless stated. MI=myocardial infarction; PVD=peripheral vascular disease; TIA=transient ischaemic attack

Table S16 Baseline characteristics of patients with different thrombotic profiles

	No marker in the top quartile (n=139)	One or two markers in the top quartile (n=120)	Three or more markers in the top quartile (n=375)	p for trend
**Age (mean/SD)**	68.3/14.2	76.3/11.7	74.3/13.2	<0.001
**Sex**	61 (43.9)	61 (50.8)	197 (52.5)	0.08
**Hypertension**	76 (54.7)	75 (62.5)	232 (61.9)	0.15
**Diabetes**	15 (10.8)	15 (12.5)	44 (11.7)	0.78
**Hyperlipidaemia**	48 (34.5)	34 (28.3)	104 (27.7)	0.14
**Atrial fibrillation**	14 (10.1)	22 (18.3)	72 (19.2)	0.02
**Current smoking**	24 (17.3)	14 (11.7)	56 (14.9)	0.55
**Chronic kidney disease**	69 (49.6)	79 (65.8)	209 (55.7)	0.28
**Previous MI**	13 (9.4)	10 (8.3)	37 (9.9)	0.84
**Previous PVD**	9 (6.5)	8 (6.7)	40 (10.7)	0.13
**Previous stroke**	16 (11.5)	14 (11.7)	60 (16.0)	0.18
**Previous TIA**	16 (11.5)	18 (15.0)	44 (11.7)	0.99

Numbers are reported as n (%) unless stated. MI=myocardial infarction; PVD=peripheral vascular disease; TIA=transient ischaemic attack

Table S17 Associations of biomarkers in the top quartile and risks of recurrent stroke and risks of myocardial infarction or sudden cardiac death, stratified by previous history of coronary artery disease

	**Inflammatory markers**		**Thrombotic markers**	
	Adjusted HR (95%CI)	p	Adjusted HR (95%CI)	p
**Recurrent stroke:**				
**Any biomarker in the top quartile vs. the rest:**				
Yes	**2.14 (1.20-3.82)**	**0.01**	1.42 (0.86-2.35)	0.17
No	1.10 (0.82-1.47)	0.53	1.20 (0.49-2.90)	0.69
**Per number in the top quartile:**				
Yes	**1.44 (1.13-1.82)**	**0.003**	1.03 (0.87-1.23)	0.72
No	1.13 (0.99-1.29)	0.08	1.09 (0.80-1.50)	0.59
**Myocardial infarction or sudden cardiac death**				
**Any biomarker in the top quartile vs. the rest:**				
Yes	**0.50 (0.24-1.01)**	**0.05**	2.24 (0.62-8.04)	0.22
No	1.36 (0.79-2.36)	0.27	1.96 (0.69-5.54)	0.21
**Per number in the top quartile:**				
Yes	0.80 (0.57-1.12)	0.19	1.24 (0.81-1.90)	0.33
No	1.15 (0.90-1.49)	0.27	1.20 (0.85-1.69)	0.30

HR=hazard ratios and were adjusted for age, sex, history of hypertension, diabetes, hyperlipidaemia, atrial fibrillation, current smoking, chronic kidney disease, previous stroke, transient ischaemic attack, peripheral arterial disease and myocardial infarction.

Table S18 Associations of clustering of inflammatory biomarkers (per number in the top quartile) and risks of recurrent stroke stratified by levels of thrombotic or neuronal markers

	Time to first event		All events	
	Adjusted HR (95%CI)	p	Adjusted IRR (95%CI)	p
**By neuronal markers**				
At least one in the top quartile	1.31 (1.10-1.56)	0.003	1.34 (1.10-1.64)	0.004
The rest	1.12 (0.94-1.33)	0.22	1.26 (1.04-1.53)	0.02
**By thrombotic markers:**				
At least one in the top quartile	1.13 (0.97-1.30)	0.11	1.24 (1.04-1.46)	0.01
The rest	1.39 (1.13-1.70)	0.002	1.37 (1.08-1.73)	0.01

HR=hazard ratios; IRR=incidence rate ratio.
All analyses were adjusted for age, sex, history of hypertension, diabetes, hyperlipidaemia, atrial fibrillation, current smoking, chronic kidney disease, previous stroke, transient ischaemic attack, peripheral arterial disease and myocardial infarction.

Table S19 Associations of clustering of biomarker (per number of biomarkers in the top quartile) and risk of recurrent stroke, myocardial infarction or sudden cardiac death in fully adjusted models

	All*		Excluding first 90 days*		TIA and minor stroke only*	
	**HR (95%CI)**	**p**	**HR (95%CI)**	**p**	**HR (95%CI)**	**p**
**Any biomarker**	1.30 (1.08-1.57)	0.005	1.36 (1.10-1.67)	0.004	1.34 (1.10-1.65)	0.004
**Inflammatory**	1.18 (1.07-1.31)	0.001	1.18 (1.05-1.32)	0.005	1.24 (1.10-1.41)	0.001
**Thrombotic**	1.13 (0.99-1.30)	0.07	1.13 (0.98-1.32)	0.10	1.13 (0.97-1.31)	0.12

*Adjusted for age, sex, history of hypertension, diabetes, hyperlipidaemia, atrial fibrillation, current smoking, chronic kidney disease, previous stroke, transient ischaemic attack, peripheral arterial disease and myocardial infarction.

Table S20 Associations of biomarkers in the top quartile and risks of recurrent stroke stratified by time to index stroke

	Inflammatory markers		Thrombotic markers	
	Adjusted HR (95%CI)	p	Adjusted HR (95%CI)	p
**Any biomarker in the top quartile vs. the rest:**				
<90 days	1.50 (0.86-2.62)	0.15	1.43 (0.55-3.69)	0.46
90 day – 1 year	1.31 (0.70-2.45)	0.40	2.84 (0.67-11.96)	0.16
1-5 years	1.24 (0.82-1.87)	0.32	1.10 (0.55-2.20)	0.79
5-10 years	1.43 (0.90-2.27)	0.13	1.34 (0.64-2.83)	0.44
**Per number in the top quartile:**				
<90 days	1.24 (0.98-1.57)	0.08	1.14 (0.83-1.58)	0.42
90 day – 1 year	1.24 (0.95-1.62)	0.11	1.34 (0.89-2.01)	0.16
1-5 years	1.22 (1.01-1.47)	0.04	1.03 (0.80-1.33)	0.81
5-10 years	1.34 (1.06-1.68)	0.01	1.02 (0.78-1.34)	0.86

Table S21 Associations of biomarkers in the top quartile and risks of recurrent stroke stratified by TOAST subtype

HR=hazard ratios and were adjusted for age, sex, history of hypertension, diabetes, hyperlipidaemia, atrial fibrillation, current smoking, chronic kidney disease, previous stroke, transient ischaemic attack, peripheral arterial disease and myocardial infarction.

	**Inflammatory markers**		**Thrombotic markers**	
	Adjusted HR (95%CI)	p	Adjusted HR (95%CI)	p
**Top quartile vs. the rest**				
Cardioembolic	1.41 (0.82-2.42)	0.21	0.85 (0.38-1.90)	0.69
Large artery disease	1.53 (0.66-3.55)	0.32	4.65 (0.55-39.33)	0.16
Small vessel disease	0.92 (0.49-1.76)	0.81	1.04 (0.41-2.61)	0.94
Cryptogenic	1.18 (0.75-1.86)	0.48	1.77 (0.78-4.03)	0.17
**Per number in the top quartile:**				
Cardioembolic	1.12 (0.91-1.39)	0.28	0.99 (0.74-1.32)	0.94
Large artery disease	1.05 (0.70-1.59)	0.81	2.26 (0.90-5.66)	0.08
Small vessel disease	1.06 (0.75-1.49)	0.73	0.91 (0.63-1.30)	0.59
Cryptogenic	1.24 (0.99-1.55)	0.06	1.12 (0.85-1.48)	0.43

Figure S1 Associations of individual biomarkers and long-term risks of recurrent stroke stratified by aetiology and excluding the first 90 days

All analyses adjusted for age, sex, history of hypertension, diabetes, hyperlipidaemia, atrial fibrillation, current smoking, chronic kidney disease, previous stroke, transient ischaemic attack, peripheral arterial disease and myocardial infarction.
Hazard ratios are presented as per SD(Ln) except for fibrinogen, VWF and Protein Z which were given as SD.
IL-6=interleukin-6; CRP=C-reactive protein; NGAL=neutrophil gelatinase-associated lipocalin; TNFR=tumour necrosis factor receptor; NSE=neurone-specific enolase; BDNF=brain-derived neurotrophic factor; VWF=von Willebrand factor antigen.

Cardioembolic events	HR	95% CI
**Inflammatory markers**		
IL-6	1.12	0.83-1.51
CRP	1.11	0.81-1.54
NGAL	1.17	0.81-1.69
TNFR-1	1.02	0.74-1.40
**Neuronal function/injury**		
NSE	1.02	0.75-1.39
BDNF	1.21	0.88-1.67
**Thrombotic markers**		
Thrombomodulin	1.03	0.74-1.43
Fibrinogen	0.95	0.69-1.31
P-selectin	0.84	0.58-1.21
D-dimer	1.05	0.77-1.44
VWF	0.79	0.55-1.13
Protein Z	0.91	0.65-1.28

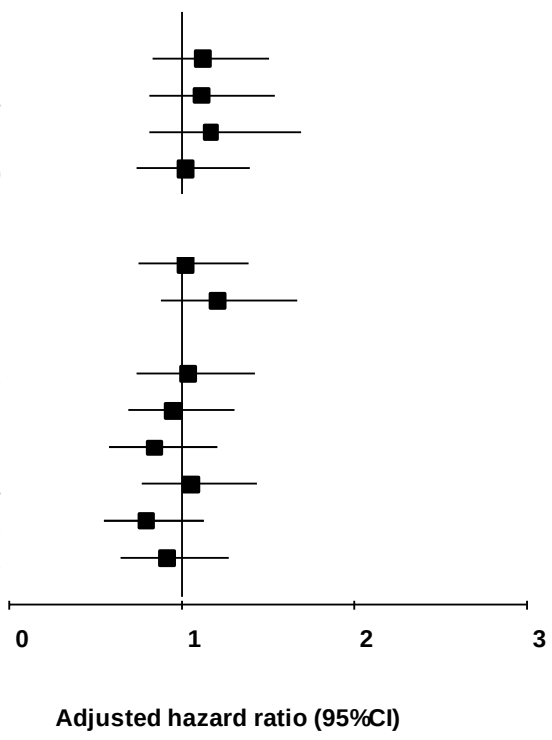

Large artery disease	HR	95% CI
**Inflammatory markers**		
IL-6	1.09	0.69-1.75
CRP	1.25	0.64-2.46
NGAL	1.58	0.84-2.98
TNFR-1	1.25	0.62-2.52
**Neuronal function/injury**		
NSE	1.46	0.85-2.53
BDNF	1.48	0.87-2.51
**Thrombotic markers**		
Thrombomodulin	0.93	0.44-1.99
Fibrinogen	11.70	0.87-157.59
P-selectin	6.17	0.58-65.75
D-dimer	2.32	0.76-7.07
VWF	0.78	0.31-1.98
Protein Z	0.68	0.27-1.69

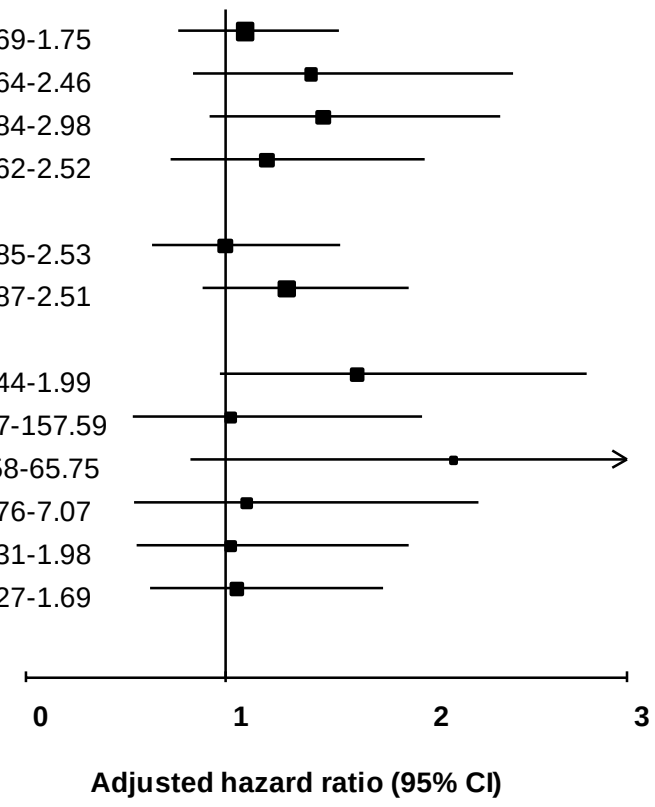

Small vessel disease	HR	95% CI
**Inflammatory markers**		
IL-6	1.00	0.69-1.45
CRP	1.06	0.74-1.52
NGAL	1.12	0.77-1.64
TNFR-1	1.10	0.73-1.66
**Neuronal function/injury**		
NSE	1.21	0.84-1.73
BDNF	0.85	0.62-1.17
**Thrombotic markers**		
Thrombomodulin	0.86	0.55-1.34
Fibrinogen	0.79	0.44-1.42
P-selectin	0.79	0.51-1.22
D-dimer	1.14	0.66-1.97
VWF	1.29	0.73-2.28
Protein Z	1.19	0.78-1.82

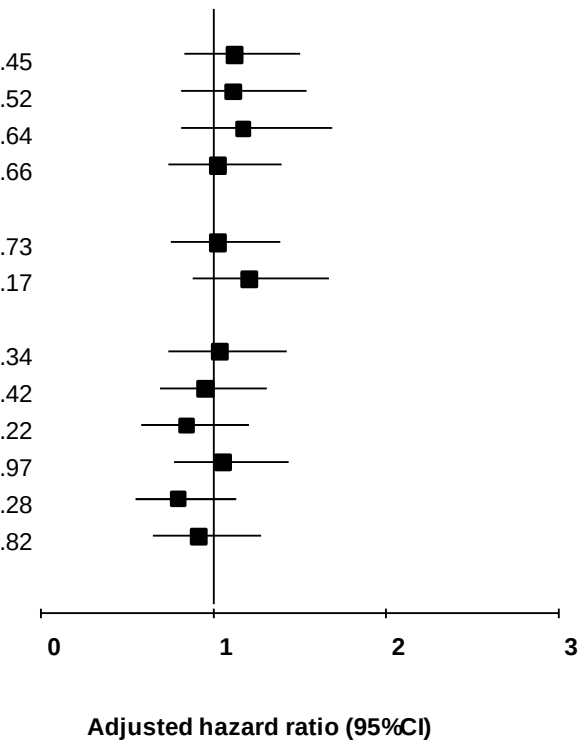

Cryptogenic events	HR	95% CI
**Inflammatory markers**		
IL-6	1.13	0.89-1.45
CRP	1.14	0.89-1.47
NGAL	0.98	0.77-1.24
TNFR-1	1.21	0.92-1.60
**Neuronal function/injury**		
NSE	1.04	0.81-1.33
BDNF	0.97	0.75-1.25
**Thrombotic markers**		
Thrombomodulin	1.00	0.77-1.30
Fibrinogen	1.06	0.71-1.58
P-selectin	1.36	0.94-1.98
D-dimer	1.55	1.08-2.22
VWF	1.16	0.79-1.71
Protein Z	1.20	0.86-1.67

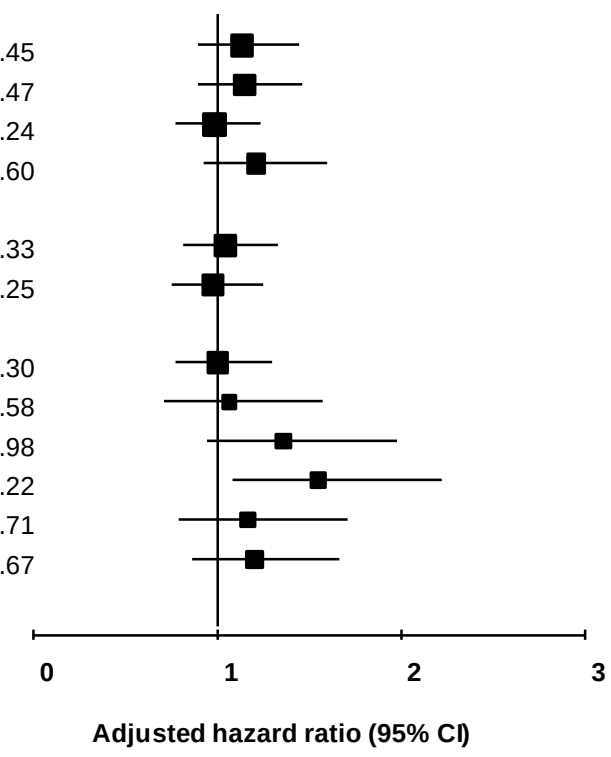

Figure S2 10-year risk of recurrent stroke in those with at least one biomarker in the top quartile vs. no biomarker in the top quartile (left panel: all patients; right panel: excluding the first 90 days)

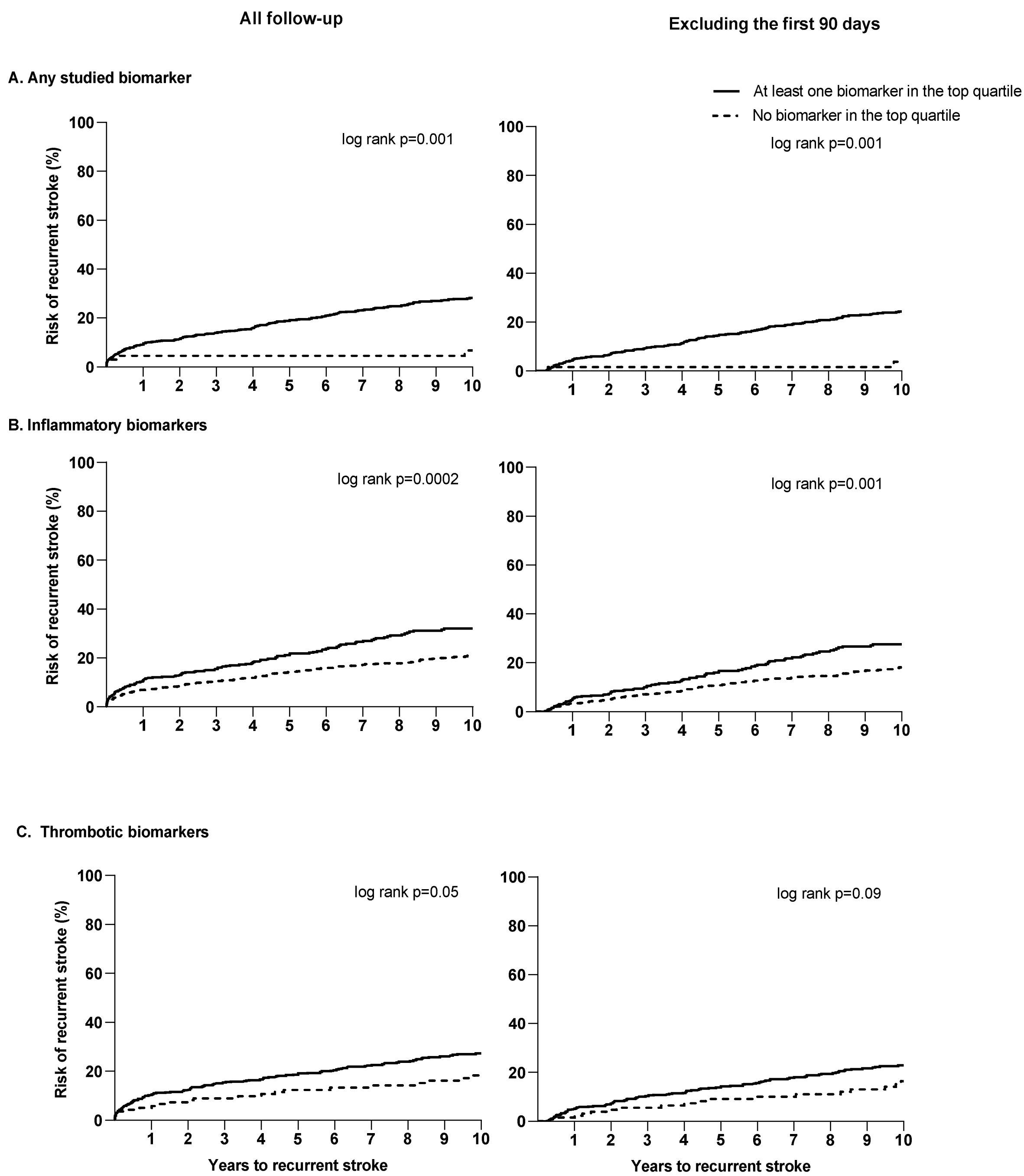

Supplement: Supplementary file 1 [file str-57-3035-s001.pdf]
